# Supplementary material for: Comorbidities in osteoarthritis (ComOA): a combined cross-sectional, case–control and cohort study using large electronic health records in four European countries
Source: BMJ Open. 2022 Apr 5;12(4):e052816. doi: 10.1136/bmjopen-2021-052816 (PMC8987784; doi:10.1136/bmjopen-2021-052816)
Supplement: Author's manuscript [file bmjopen-2021-052816.draft_revisions.pdf]

BMJ Open is committed to open peer review. As part of this commitment we make the peer review history of every article we publish publicly available.

When an article is published we post the peer reviewers' comments and the authors' responses online. We also post the versions of the paper that were used during peer review. These are the versions that the peer review comments apply to.

The versions of the paper that follow are the versions that were submitted during the peer review process. They are not the versions of record or the final published versions. They should not be cited or distributed as the published version of this manuscript.

BMJ Open is an open access journal and the full, final, typeset and author-corrected version of record of the manuscript is available on our site with no access controls, subscription charges or pay-per-view fees (http://bmjopen.bmj.com).

If you have any questions on BMJ Open's open peer review process please email info.bmjopen@bmj.com

BMJ Open

Comorbidities in Osteoarthritis (CoMO): a multinational study in four European countries

Journal:	*BMJ Open*
Manuscript ID	bmjopen-2021-052816
Article Type:	Protocol
Date Submitted by the Author:	26-Apr-2021
Complete List of Authors:	Swain, Subhashisa; University of Nottingham, Academic Rheumatology<br>Kamps, Anne; Erasmus MC - University Medical Center Rotterdam, Department of General Practice<br>Runhaar, Jos; Erasmus University Medical Center Rotterdam, Department of General Practice<br>Dell'Isola, Andrea; Lunds University Faculty of Medicine,<br>Turkiewicz, Aleksandra; Lund University, Dept of Orthopedics<br>Robinson, Danielle ; University of Oxford Nuffield Department of Orthopaedics Rheumatology and Musculoskeletal Sciences,<br>Strauss, V; University of Oxford Nuffield Department of Orthopaedics Rheumatology and Musculoskeletal Sciences,<br>Mallen, Christian; Keele University, Arthritis Research UK Primary Care Centre<br>Kuo, Chang-Fu; Chang Gung Memorial Hospital Linkou Branch,<br>Coupland, Carol; University of Nottingham, Division of Primary Care<br>Doherty, Michael ; University of Nottingham, Academic Rheumatology<br>Sarmanova, Aliya ; University of Bristol,<br>Prieto-Alhambra, Daniel; University of Oxford, Centre for Statistics in Medicine, NDORMS<br>Englund, Martin; Lund University, Dept of Orthopedics<br>Bierma-Zeinstra, Sita; Erasmus University Medical Centre, Department of General Practice<br>Zhang, Weiya; University of Nottingham, Academic Rheumatology
Keywords:	EPIDEMIOLOGY, Musculoskeletal disorders < ORTHOPAEDIC & TRAUMA SURGERY, RHEUMATOLOGY

SCHOLARONE™
Manuscripts

Title- Comorbidities in Osteoarthritis (CoMO): a multinational study in four European countries

Authors

Subhashisa Swain[1,10], Anne Kamps[2], Jos Runhaar[2], Andrea Dell 'Isola[3], Aleksandra Turkiewicz[3], Danielle Robinson[4], Victoria Y Strauss[4], Christian Mallen[5], Chang Fu Kuo[1,6], Carol Coupland[7], Michael Doherty[1,10], Aliya Sarmanova[8], Daniel Prieto Alhambra[4], Martin Englund[3], Sita Bierma-Zeinstra[9], Weiya Zhang[1,10]

1. Academic Rheumatology, School of Medicine, University of Nottingham, UK
2. Department of General Practice, Erasmus MC - University Medical Center Rotterdam, The Netherlands
3. Clinical Epidemiology Unit, Orthopaedics, Department of Clinical Sciences Lund, Lund University, Sweden
4. Centre for Statistics in Medicine, NDORMS, University of Oxford, UK
5. School of Medicine, Keele University, UK
6. Division of Rheumatology, Allergy and Immunology, Chang Gung Memorial Hospital, Taiwan.
7. Division of Primary Care, School of Medicine, University of Nottingham, UK
8. Musculoskeletal Research Unit, Bristol Medical School, Translational Health Sciences, University of Bristol, UK
9. Department of General Practice, Department of Orthopaedic Surgery, Erasmus MC - University Medical Center Rotterdam, The Netherlands
10. Pain Centre Versus Arthritis, University of Nottingham, UK

Corresponding Author:

Professor Weiya Zhang
Academic Rheumatology, School of Medicine
University of Nottingham
Clinical Sciences Building, Nottingham city Hospital
NG5 1PB, Nottingham, UK
Email: weiya.zhang@nottingham.ac.uk

For peer review only - http://bmjopen.bmj.com/site/about/guidelines.xhtml

Abstract

Introduction

Osteoarthritis (OA) is one of the leading chronic conditions in the older population. People with OA are more likely to have one or more other chronic conditions than those without. However, the temporal associations, clusters of the comorbidities, role of analgesics and the causality and variation between populations are yet to be investigated. This paper describes the protocol of a multinational study in four European countries (UK, Netherlands, Sweden, and Spain) exploring comorbidities in people with OA .

Methods and analysis

This multinational study will investigate i) the temporal associations of 61 identified comorbidities with OA, ii) the clusters and trajectories of comorbidities in people with OA, iii) the role of analgesics on incidence of comorbidities in people with OA, iv) the potential biomarkers and mechanistic pathways between OA and the comorbidities, and v) variations between countries.

A combined case-control and cohort study will be conducted to find the temporal association of OA with the comorbidities using the national or reginal health databases. Latent class analysis will be performed to identify the clusters at baseline and joint latent class analysis will be used to examine trajectories during the follow-up. A cohort study will be undertaken to evaluate the role of non-steroidal anti-inflammatory drugs (NSAIDs), opioids, and paracetamol on the incidence of comorbidities. Mendelian randomisation will be performed to investigate the potential biomarkers for causality between OA and the comorbidities using the UK Biobank and the Rotterdam databases. Finally, a meta-analysis will be used to examine the variations and pool the results from different countries.

Ethics and Dissemination

Research ethics was obtained according to each database requirement. Results will be disseminated through the FOREUM website, scientific meetings, publications, and in partnership with patient organisations.

Key words: Osteoarthritis; Comorbidity; Multimorbidity; Primary care; Analgesics, Mendelian Randomisation, PheWAS

For peer review only - http://bmjopen.bmj.com/site/about/guidelines.xhtml

Strengths and limitations of this study

- This is first ever multicenter study on comorbidities in osteoarthritis in Europe involving nearly 27 million electronic health records
- More than 60 chronic conditions are being studied – representing a wider coverage of diseases
- We will examine the causal association using Mendelian Randomization -PheWAS methods with genetic data collected from two countries
- Same protocol with robust statistical methods will be used across all countries to replicate the findings and examine the variations.
- Possible biases may be introduced by the nature of electronic health records and length of data availability.

For peer review only

For peer review only - http://bmjopen.bmj.com/site/about/guidelines.xhtml

Background

Osteoarthritis (OA) affects 27% of people aged over 45 years at peripheral synovial joints such as knees, hips, hands and feet (1) and it is by far the most common arthritis and a leading cause of chronic joint pain and disability in older people (2,3). It is anticipated that the burden of OA will continue to rise in the coming decades because of aging and obesity – two major risk factors for OA (4,5). A recent systematic review has confirmed that people with OA are more likely to have other diseases, especially stroke, peptic ulcer, hypertension, and depression (6). However, whether these comorbidities just co-exist with OA, share common risk factors with OA, or are causes or consequences of OA remains largely unknown.

There was wide heterogeneity in definitions of OA and other chronic conditions, diagnosis and recording of diseases, sample sizes, and number of diseases studied (6). This diversity made the comparison and pooled estimation of comorbidity prevalence difficult. One of the major limitations of previous small studies was, they mostly focused on cardiovascular and musculoskeletal conditions (7–9). Various hypotheses have been used to explain the existence of comorbidities in general, the most accepted of which are the concordant (diseases sharing similar pathophysiological risk factors) and discordant (diseases not sharing similar pathophysiological risk factors) theories (10). Multiple factors are reported to be associated with comorbidity and multimorbidity, and prescription of drugs is one of these (11). That is the presence of multiple chronic conditions increases the chances of polypharmacy which further escalates the risk of other conditions. Especially in people with OA, the prescription of analgesics is common, and is associated with increased risk of other conditions such as cardiovascular, gastrointestinal and chronic renal diseases (12,13). The additional presence of disease has a significant impact on an individual. OA is one of the leading conditions reported in multimorbidity research and exploring the association with other diseases would help in further explaining the causality (14). Understanding the temporal association and disease trajectory is crucial for any chronic condition, and this is possible through studies using longitudinal databases (15). Observational studies have limitations of establishing causal association between OA and comorbidities, for which newly developed 'Mendelian Randomisation' method is more powerful.

Another major issue in comorbidity research is the number and types of conditions studied (16). Therefore, it is important to develop a consensus on both the count and typology of conditions to be studied to enable comparisons across populations and to derive pooled estimates as appropriate. Further, using uniform methods and definitions of diseases in

For peer review only - http://bmjopen.bmj.com/site/about/guidelines.xhtml

computing these estimates would reduce heterogeneity and make the comparison more reliable. The burden of diseases in primary care often depends on the population structure, health infrastructure and individual factors such as income and education. Such factors vary between countries, but because of the heterogeneity mentioned above there are no robust data comparing OA and its comorbidities between countries. Therefore, the aims of this study were to explore the burden, pattern, and causal factors of comorbidities in people with OA across four European countries using national registration databases in the UK, the Netherlands, Sweden, and Spain.

For peer review only

Objectives

1. To estimate the prevalence, incidence, and time sequence of comorbidities in OA
2. To examine the clusters of comorbidities and trajectories of clusters in OA and associations with death
3. To investigate the associations between commonly used OA drugs, such as non-steroidal anti-inflammatory (NSAIDs) and opioids, and risk of comorbidities
4. To identify the potential biomarkers and mechanistic pathways between OA and the comorbidities
5. To examine the variations of OA comorbidities and clusters across countries.

Methods

Ethics

The study has obtained the following ethics approvals: UK- ISAC 19/30R, Netherlands IPCI registration no. 11/2019, Spain - 4R19/011, Sweden – Dnr 2011-432, Dnr 2014-276, and Dnr 2018-233.

Databases

Four routinely collected national (the UK and the Netherlands) or regional (Sweden and Spain) health databases will be used for objectives 1-3. In addition, for objective 4, genomic associations of OA with comorbidities will be examined using two cohort studies from the UK (UK Biobank) (17) and the Netherlands (Rotterdam study) (18). The four national representative and regional databases contain information about the population with primary care consultations in four different countries. The longitudinal databases provide information about the diagnosis of the diseases by the general practitioners and some diagnoses made in secondary care, prescription of drugs, deaths, and other health utilisation indicators. The details of the databases and their properties are given in Table 1 and 2.

For peer review only - http://bmjopen.bmj.com/site/about/guidelines.xhtml

Participants
People registered with the respective databases aged 18 year or above are eligible for the study.

Patient public involvement (PPI): Three PPI representatives (with OA and all with multiple chronic conditions) were involved in this study through group meetings. Difficulties of living with multiple conditions, lack of research in causal relationship and identification of diseases to be studied and the role of drugs in comorbidity were discussed. They are constantly in touch through providing their inputs at each steps of the study.

Definition of Osteoarthritis
OA will be defined as the at least one recorded physician diagnosis of OA for hip, knee, ankle/foot, wrist/hand, or site recorded as 'unspecified' during the study period for the respective database. People with any previous recording of the OA prior to the first diagnosis (index date) will be excluded.

Comorbidities
We defined comorbidity as the recording of diagnosis of predefined chronic conditions in individuals using either ICD-10 or Read or international classification of primary care (ICPC) code. An extensive list of 61 chronic conditions was prepared from the Quality Outcome Framework (QOF) (19), list of the US Department of Health and Human Services Initiative on Multiple Chronic Conditions (20), global burden of diseases (21) and the Charlson comorbidity index (22). The list has been updated with findings from our systematic review (6) and a previous UK community-based knee pain study (6,23) by including common and important morbidities not included in the above (24,25). A code mapping exercise was conducted to finalise the list of conditions available for all the research centres. The comparison of codes was made, and it was reviewed by four researchers including a clinician from the team. The detailed list of the conditions is given in Table 3.

Covariates
Age, and gender will be used in all centres as covariates to adjust in regression models. Additionally, information on body mass index (BMI), smoking, alcohol use, socioeconomic variables such as education level, income, place of birth (to identify those who immigrated to the country), and residential area, marriage (or registered partner) will be included when available. For calculating severity of the comorbidities in an individual, Elixhauser comorbidity index will be used to estimate the impact of comorbidities on death (26,27).

For peer review only - http://bmjopen.bmj.com/site/about/guidelines.xhtml

Missing data on covariates will be substituted using multiple imputation methods, provided that the data is missing at random, if applicable.

Study design and data analysis

Summary of the study design and analysis is provided in Figure 1.

Objective 1 – Prevalence, incidence, and time sequence of comorbidities in OA

A combined retrospective and prospective study of OA cases and sex, age (+/-2 years), first year of registration, and practice matched controls (1:1-4) without OA (28) will be used to determine the prevalence, incidence, and time sequence of comorbidities in OA. Incident OA cases will be identified, and the first diagnosis date will be used as the starting point (index date). For controls the same index date as their matched case will be used. They will be both retrospectively reviewed for prior diagnoses of comorbidities and prospectively followed-up for posterior new comorbidities. In the retrospective analysis the prevalence and 95% confidence interval (CI) of each specific comorbidity will be calculated separately in OA cases and matched controls using the number of people diagnosed with the comorbidity divided by the total number of OA cases or controls at the index date. The prevalence of each comorbidity in OA cases and matched controls will be calculated for given time intervals prior to the index date of 0-1, 0-5 and 0-10 years separately to assess observational bias (28). Discrete time intervals of 1-5, and 5-10 years before will also be used to estimate the prevalence to minimise consultation bias/misclassification bias of OA (if possible). Conditional logistic regression will be used to calculate the odds ratios (OR) for each comorbidity unadjusted and adjusted for BMI, smoking and alcohol consumption.
For the prospective analysis participants with incident OA but without the specific comorbidity of interest at the index date (i.e., people at risk) and matched controls without OA will be followed up until the date of the first diagnosis of the comorbidity, deregistration, or death whichever comes first. The cumulative incidence will be calculated for each comorbidity in OA cases and matched controls at 1, 3, 5, 10, 15, 20 years (based on the data available) after the index date to examine the dynamic change of developing comorbidities during follow-up. Kaplan-Meier survival curves will be used to display the cumulative probability in OA and non-OA groups. Proportional hazard assumption will be tested using Schoenfeld residual plots. The Cox regression model will be used to calculate hazard ratios (HR) for each comorbidity unadjusted and adjusted for age, gender, practice, BMI, smoking

For peer review only - http://bmjopen.bmj.com/site/about/guidelines.xhtml

and alcohol consumption. This hybrid design has been previously used by us to examine the temporality of associations between other rheumatic musculoskeletal diseases (RMDs) (e.g., gout and lupus) and comorbidities (28,29).

Objective 2 – Clusters and impact of comorbidities in people with OA

For each dataset, an 80%: 20% split into the training and testing data will be introduced. The following analysis in objective 2 will be first employed into the training dataset and then tested its generalisability in the testing dataset. At baseline, clusters of people based on 61 comorbidities will be identified using Latent class (i.e., Gaussian mixture models algorithms of cluster) analysis (30). For each model, we will examine the association between clusters and covariates using multinomial logistic regressions. The distinctness of clusters will be examined by comparing covariates among clusters. The optimal model is the one where most clusters found in the training data are also identified independently in the testing data and clusters have most distinct patients' characteristics. We will then use both latent trajectory analysis, such as joint latent class models (31), and unsupervised machine learning approach, such as deep autoencoder or recurrent neural networks (32), to identify distinct clusters of new comorbidity numbers development over time and their association with mortality with adjustment for baseline covariates.

Objective 3 - Association between OA drugs and incident comorbidities

A cohort study will be undertaken for this objective to evaluate the contribution of common analgesics for OA to the development of comorbidity such as NSAIDs, opioids and paracetamol. We are interested in the interaction between OA and use of drugs on the incidence of comorbidities, i.e. to evaluate if the drug use in persons with OA poses increased or decreased risk of comorbidities compared to persons without OA and/or analgesics. Individual comorbidity, as well as clusters of comorbidities identified from Objective 2 will be examined as outcomes. The 61 comorbidities in our study will be further categorised into eight groups, specifically: musculoskeletal (MSK), respiratory, neurodegenerative, psychological/psychiatric, cancer, cardiovascular, metabolic, renal problem, liver diseases, gastrointestinal (GI) and others. (Table 4) The prospective cohort established from Objective 1 will form the source population for this objective. Individuals with incident OA will be identified from the database and the first diagnosis date will be used as index date for follow-up. Individuals without OA during the study period will be selected and matched with cases by age, sex, and practice. The same index date will be given from their matched OA cases.

For peer review only - http://bmjopen.bmj.com/site/about/guidelines.xhtml

Individuals with analgesics prescriptions prior to the index date will be excluded (or recorded as a confounding factor to be adjusted as appropriate). Only analgesic prescriptions after the index date will be considered for this analysis. Prescriptions will be quantified as number of prescriptions within year 1 (initial use, primary analysis) (33), 2, 3, 4, 5 etc. It will also be dichotomised as episodic (e.g., at least one gap of ≥90 days between prescriptions) and continuous (no gap of more less than 90 days) users as appropriate (34,35). Analgesic use will be included in the model as a risk factor together with OA diagnosis (yes/no, primary exposure) to examine the independent risk of each variable (OA and analgesics), as well as the interaction between the two to the development of comorbidity. Dose response relationship will be examined using number of prescriptions during the exposure window examined. The effect of stopping analgesics will also be examined by looking into the patterns of analgesic prescriptions, e.g., stopping analgesics after initial use in year 1 versus continuous use of analgesics afterwards. For the primary analysis (initial prescriptions within year 1), a landmark analysis will be used to minimise the immortal time bias where the follow up will start after 12 months from the index date (36). Participants at risk (i.e., without a specific comorbidity of interest) at the landmark date will be followed up until the first diagnosis of the comorbidity, deregistration, or death whichever comes first. For secondary analyses, time varying covariate analysis will be used to examine the long-term, episodic/continuous use of analgesics after the index date and interaction between OA and analgesics in the development of the comorbidity. The propensity score matching or the inverse probability weighing methods will be used to adjust for confounding by indication during the follow-up as appropriate. Depending on the country-specific drug use patterns, we may modify this definition to allow for short brakes in between the episodes. Cox-regression model will be used to calculate the HR and 95% CI. We will use flexible parametric models using restricted cubic splines (developed by Lambert, “stpm2” in Stata) to estimate the HRs and differences in time to diagnosis of comorbidities (outcome) with drugs as time-varying to account for non-proportional hazards (37).

Objective 4 – Potential causal pathways between OA and the comorbidities

We will perform a Mendelian Randomisation (MR) phenome-wide association (MR-PheWAS) study(38) to examine the causal relationship between OA, its phenotypes, biomarkers or risk factors and comorbidities using the UK Biobank and the Rotterdam Study database.

For peer review only

We will use the Rotterdam Study and the UK Biobank jointly for this objective. This is because that the Rotterdam Study is an OA cohort with deep phenotypes and biomarkers of OA, whereas the UK Biobank is a primary cohort for cancer and multiple disease outcomes, and both have detailed genetic variants. We will use two sample MR approach, i.e., to establish an association between OA and genetic variants in the Rotterdam Cohort to identify genetic instrumental variables (IV), e.g., a set of single nucleotide polymorphisms (SNPs) associated with OA (or a deep phenotype, biomarker, or risk factor of OA). We will then undertake the MR-PheWAS analysis to examine the causal effects of the OA IV on comorbidities in the UK Biobank. The MR method has been widely used in real world data to examine the causal relationship between IV and specific disease, under two assumptions: [1] genetic variants are randomly assigned in the population; and [2] genetic variants can only be the cause not consequence of disease (39). The PheWAS is a series of case control studies to estimate the associations between the IV and multiple disease outcomes (38,40). The combination of the two permits investigation of the causal effects of OA on multiple disease outcomes.

The MR-PheWAS analysis includes three steps. Firstly, we will identify the genetic variants that are associated with OA - IV. Secondly, we will undertake the PheWAS analysis – a series of case control analyses to estimate the associations between the IV and other disease outcomes (38,40) , with an adjustment for multiple testing using the false discovery rate (FDR) methods (41). Thirdly, we will implement conventional MR analysis to investigate the causal effects of the OA IV on comorbidities (39). An inverse variance weighted (IVW) method will be used to pool the associations (ORs) as appropriate (42). The MR-Egger regression analysis will be used to count for the pleiotropic effect - the effects of one genetic variant on multiple outcomes (43). The heterogeneity in dependent instruments (HEIDI) test will be used to exclude the cross-phenotype associations caused by genetic linkage (44).

With the MR-PheWAS study, the OR can be interpreted as causal association. We are primarily interested in the causality from OA to comorbidities. We are also interested in inflammatory (e.g., CRP), metabolic (e.g., gut microbiome) and biomechanics (e.g., BMI) biomarkers and deep phenotypes of OA such as knee, hip, and hand OA with and without symptoms. This will be undertaken if it is feasible within 3 years of this funded project, otherwise will be considered as our future research agenda.

Objective 5 – Variation of OA comorbidity patterns across countries

For peer review only - http://bmjopen.bmj.com/site/about/guidelines.xhtml

We will use meta-analysis (MA) to examine the variation between countries and to pool the data as appropriate. Estimates from first three objectives such as prevalence, incidence, OR, HR and 95% CI for each specific comorbidity across different populations will be distributed in a forest plot. Heterogeneity will be examined using the I^2 statistic and the Q test (45). Results will be pooled if they are homogenous based on the I^2 value using the fixed effects model, otherwise the reasons for the heterogeneity will be investigated. Random effects models will be used to pool the results if the reasons for the heterogeneity cannot be identified and if the overall pooling is appropriate. Individual patient data (IPD) meta-analysis may be used to help identify the reasons for heterogeneity (46). Common clusters and trajectories as well as burdens of comorbidities will also be compared between populations.

Discussion

OA is the most common arthritis and the second most common musculoskeletal (the first being back pain) in older people. However, unlike other RMDs, relatively little is known about comorbidities in OA. OA previously was defined as a “wear and tear” joint-specific degenerative condition, but recent research has found that it is a common complex disorder which may increase the risk of other chronic conditions in other systems. This study will be the largest epidemiological study on comorbidities of OA in primary care.

One of the key advantages of this multinational study is the use of the same protocol to measure the burden of comorbidities in primary care settings in four European countries to ensure reproducibility and comparison. There is scant evidence on the comorbidities in people with OA, and this approach should help to identify the leading and most important associations before and after presenting clinical OA (the index date). Further advantages of this study are the large and representative populations studied and the same/similar extensive list of chronic conditions for identifying comorbidity clusters. Often comorbidities accumulate with age over time and the large primary care databases in this study have the advantage of having long follow-up time which will enable us to detect the incidence of comorbidities. Also, longer follow up would help to identify the picture of the trajectory of the diseases (47). Both the incidence and the trajectories of comorbidity clusters are highlighted as key elements needed in current research in multimorbidity, so findings from this study should help to fill the knowledge gaps on multimorbidity in OA.

The relationship between chronic conditions and polypharmacy is a complex area of research. The count of the medications and more importantly the nature of prescribed drugs may be

For peer review only - http://bmjopen.bmj.com/site/about/guidelines.xhtml

For peer review only

responsible for developing many new comorbidities in people with OA. We aim to explore the associations of the most commonly prescribed drugs in OA, such as NSAIDs, with the incidence of a wide range of comorbidities, which will be the first time that conditions other than established comorbidities such as psychological conditions and endocrine diseases will be examined. Finally, the causality study will further explore the associations at genetic levels and phenotypes, which will be novel in OA research. Using a two sample MR approach - one for OA deep phenotypes and the other for other chronic conditions maximises the potentials of sample size, disease phenotypes and comorbidity spectrum to better explore the causal pathways between OA and comorbidity.

There are some limitations to this study. Firstly, there are inherent issues in the nature of electronic health records with respect to possible misdiagnosis, ascertainment biases, under- or over-recording, and changes in databases due to change in coding structures. Also, the analysis will be restricted to fewer covariates in some databases due to missing information on lifestyle factors such as physical activities and diet. Even though the databases have different durations of data available, if possible we will use a common follow-up time for objective five.

Chronic conditions, especially comorbidities recorded in general practices, depend on multiple factors such as population structure, health care facilities, health policies, and the nature of the national databases. A major strength of this study is that it will include medical records on approximately 27 million people in four European countries. Also, the study will cover the sequence of research questions in comorbidity or multimorbidity starting from the burden through to the causality and variation. Such a research model can be used for other similar multimorbidity studies. The expected results should inform health professionals in primary care settings with respect to management of people with OA and associated comorbidities.

Status of the study

All the centres have obtained the necessary approvals for using the database in 2020. A consensus has been made on the code mapping exercise. The statistical analysis will be explained in detail in each of the publications. The team is expected to produce results by mid-2021.

Acknowledgements: We thank the Patient Research Participants (PRP) members Jenny Cockshull, Stevie Vanhegan, and Irene Pitsillidou for their involvement since the beginning of the project. We would like to thank the FOREUM for financially supporting the research. The authors would like to acknowledge Keele University's Prognosis and Consultation

For peer review only - http://bmjopen.bmj.com/site/about/guidelines.xhtml

Epidemiology Research Group who have given us permission to utilise the Code Lists (©2014).

Contributor and guarantor information:

WZ, MD, CC, SMA, ME, DA conceived and designed the study. SS, AK, AD, AT, DR and will perform the analysis and CC, WZ, JR and AT will supervise the statistical analysis. All authors contributed to the critical revision of the manuscript for important intellectual content. The corresponding author attests that all listed authors meet authorship criteria and that no others meeting the criteria have been omitted.

Funding: This work was supported by Foundation for Research in Rheumatology (FOREUM) grant (2019-2022), The Swedish Research Council (2020-01103), Governmental funding of clinical research within the national health services (ALF), and The Swedish Rheumatism Association. CM is funded by the National Institute for Health Research (NIHR) Applied Research Collaboration West Midlands, the National Institute for Health Research (NIHR) School for Primary Care Research and a National Institute for Health Research (NIHR) Research Professorship in General Practice (NIHR-RP-2014-04-026)

Role of the funding sources: The sponsors did not participate in the design and conduct of the study; collection, management, analysis, and interpretation of the data; or preparation, review, or approval of the manuscript and the decision to submit the manuscript for publication.

Competing interests: WZ declares serving as an advisory board for Ely Lilly (Ixekizumad, 2020) and Regeneron (Fasinomab, 2020). ME declares serving as an advisory Panel Board Member for Pfizer (Nov 2019, Tanezumab). CM provided advice to BMS on recruiting to a non-pharmacological atrial fibrillation trial. The other authors report no financial competing interests.

Copy right: The Corresponding Author has the right to grant on behalf of all authors and does grant on behalf of all authors, an exclusive licence (or non-exclusive for government employees) on a worldwide basis to the BMJ Publishing Group Ltd to permit this article (if accepted) to be published in BMJ editions and any other BMJPGL products and sublicences such use and exploit all subsidiary rights, as set out in our licence.

Studies involving humans or animals: No direct participant recruitment was done for the study.

For peer review only - http://bmjopen.bmj.com/site/about/guidelines.xhtml

Data sharing statement: We used anonymised data on individual patients on which the analysis, results, and conclusions reported in the paper are based. The used data is not distributable under licence. However, the relevant data can be obtained directly from the respective agencies. The codes developed for the analysis can be available upon a valid request.

For peer review only

For peer review only - http://bmjopen.bmj.com/site/about/guidelines.xhtml

Reference

1. Turkiewicz A, Petersson IF, Björk J, Hawker G, Dahlberg LE, Lohmander LS, et al. Current and future impact of osteoarthritis on health care: a population-based study with projections to year 2032. Osteoarthritis Cartilage. 2014 Nov;22(11):1826–32.

2. Badley EM. The effect of osteoarthritis on disability and health care use in Canada. J Rheumatol Suppl. 1995 Feb;43:19–22.

3. McDonough CM, Jette AM. The Contribution of Osteoarthritis to Functional Limitations and Disability. Clin Geriatr Med. 2010 Aug;26(3):387–99.

4. Cieza A, Causey K, Kamenov K, Hanson SW, Chatterji S, Vos T. Global estimates of the need for rehabilitation based on the Global Burden of Disease study 2019: a systematic analysis for the Global Burden of Disease Study 2019. The Lancet. 2020 Dec;396(10267):2006–17.

5. World Health Organization. Musculoskeletal conditions [Internet]. [cited 2021 Apr 6]. Available from: https://www.who.int/news-room/fact-sheets/detail/musculoskeletal-conditions

6. Swain S, Sarmanova A, Coupland C, Doherty M, Zhang W. Comorbidities in Osteoarthritis: A systematic review and meta-analysis of observational studies. 2019 Jun 17 [cited 2019 Jun 30]; Available from: http://doi.wiley.com/10.1002/acr.24008

7. Suri P, Morgenroth DC, Hunter DJ. Epidemiology of osteoarthritis and associated comorbidities. PM R. 2012 May;4(5 Suppl):S10-19.

8. Hall AJ, Stubbs B, Mamas MA, Myint PK, Smith TO. Association between osteoarthritis and cardiovascular disease: Systematic review and meta-analysis. Eur J Prev Cardiol. 2016;23(9):938–46.

9. Stubbs B, Aluko Y, Myint PK, Smith TO. Prevalence of depressive symptoms and anxiety in osteoarthritis: a systematic review and meta-analysis. Age Ageing. 2016 Mar 1;45(2):228–35.

10. Ricci-Cabello I, Stevens S, Kontopantelis E, Dalton ARH, Griffiths RI, Campbell JL, et al. Impact of the Prevalence of Concordant and Discordant Conditions on the Quality of Diabetes Care in Family Practices in England. Ann Fam Med. 2015 Nov 1;13(6):514–22.

11. Calderón-Larrañaga A, Poblador-Plou B, González-Rubio F, Gimeno-Feliu LA, Abad-Díez JM, Prados-Torres A. Multimorbidity, polypharmacy, referrals, and adverse drug events: Are we doing things well? Br J Gen Pract. 2012;62(605):e821–6.

12. Jüni P, Nartey L, Reichenbach S, Sterchi R, Dieppe PA, Egger M. Risk of cardiovascular events and rofecoxib: cumulative meta-analysis. The Lancet. 2004 Dec;364(9450):2021–9.

13. Castellsague J, Riera-Guardia N, Calingaert B, Varas-Lorenzo C, Fourrier-Reglat A, Nicotra F, et al. Individual NSAIDs and Upper Gastrointestinal Complications: A Systematic Review and Meta-Analysis of Observational Studies (the SOS Project). Drug Saf. 2012 Dec;35(12):1127–46.

14. Lowe D.B., Taylor M.J., Hill S.J. Cross-sectional examination of musculoskeletal conditions and multimorbidity: influence of different thresholds and definitions on prevalence and association estimates. BMC Res Notes. 2017;10(1):51.

15. Alaeddini A., Jaramillo C.A., Faruqui S.H.A., Pugh M.J. Mining Major Transitions of Chronic Conditions in Patients with Multiple Chronic Conditions. Methods Inf Med. 2017;56(5):391–400.

For peer review only - http://bmjopen.bmj.com/site/about/guidelines.xhtml

16. John R, Kerby DS, Hagan Hennessy C. Patterns and Impact of Comorbidity and Multimorbidity Among Community-Resident American Indian Elders. The Gerontologist. 2003 Oct 1;43(5):649–60.

17. Sudlow C, Gallacher J, Allen N, Beral V, Burton P, Danesh J, et al. UK Biobank: An Open Access Resource for Identifying the Causes of a Wide Range of Complex Diseases of Middle and Old Age. PLOS Med. 2015 Mar 31;12(3):e1001779.

18. Engelhart MJ, Geerlings MI, Meijer J, Kiliaan A, Ruitenberg A, van Swieten JC, et al. Inflammatory Proteins in Plasma and the Risk of Dementia: The Rotterdam Study. Arch Neurol. 2004 May 1;61(5):668.

19. Quality and Outcome Framework (QOF) [Internet]. [cited 2018 Mar 21]. Available from: https://digital.nhs.uk/article/8910/Quality-and-Outcome-Framework-QOF-Indicators-No-Longer-In-QOF-INLIQ-Enhanced-Services-ES-Vaccinations-and-Immunisations-V-I-and-GMS-Core-Contract-CC-extraction-specifications-business-rules-

20. Medicare C for, Baltimore MS 7500 SB, Usa M. CC_Main [Internet]. 2017 [cited 2018 Oct 24]. Available from: https://www.cms.gov/Research-Statistics-Data-and-Systems/Statistics-Trends-and-Reports/Chronic-Conditions/CC_Main.html

21. James SL, Abate D, Abate KH, Abay SM, Abbafati C, Abbasi N, et al. Global, regional, and national incidence, prevalence, and years lived with disability for 354 diseases and injuries for 195 countries and territories, 1990–2017: a systematic analysis for the Global Burden of Disease Study 2017. The Lancet. 2018 Nov;392(10159):1789–858.

22. Charlson ME, Pompei P, Ales KL, MacKenzie CR. A new method of classifying prognostic comorbidity in longitudinal studies: Development and validation. J Chronic Dis. 1987 Jan 1;40(5):373–83.

23. Sarmanova A, Fernandes GS, Richardson H, Valdes AM, Walsh DA, Zhang W, et al. Contribution of central and peripheral risk factors to prevalence, incidence and progression of knee pain: a community-based cohort study. Osteoarthritis Cartilage. 2018 Nov;26(11):1461–73.

24. Charlson M, Szatrowski TP, Peterson J, Gold J. Validation of a combined comorbidity index. J Clin Epidemiol. 1994 Nov 1;47(11):1245–51.

25. Quan H, Li B, Couris CM, Fushimi K, Graham P, Hider P, et al. Updating and Validating the Charlson Comorbidity Index and Score for Risk Adjustment in Hospital Discharge Abstracts Using Data From 6 Countries. Am J Epidemiol. 2011 Mar 15;173(6):676–82.

26. van Walraven C, Austin PC, Jennings A, Quan H, Forster AJ. A Modification of the Elixhauser Comorbidity Measures Into a Point System for Hospital Death Using Administrative Data: Med Care. 2009 Jun;47(6):626–33.

27. Elixhauser A, Steiner C, Harris DR, Coffey RM. Comorbidity measures for use with administrative data. Med Care. 1998 Jan;36(1):8–27.

28. Kuo C-F, Grainge MJ, Mallen C, Zhang W, Doherty M. Comorbidities in patients with gout prior to and following diagnosis: case-control study. Ann Rheum Dis. 2014 Nov 14;annrheumdis-2014-206410.

29. Swain S, Coupland C, Mallen C, Kuo CF, Sarmanova A, Bierma-Zeinstra SMA, et al. Temporal relationship between osteoarthritis and comorbidities: a combined case control and cohort study in the UK primary care setting. Rheumatology. 2021 Mar 1;keab067.

For peer review only

30. Collins LM, Lanza ST. Latent Class and Latent Transition Analysis [Internet]. Hoboken, NJ, USA: John Wiley & Sons, Inc.; 2009 [cited 2020 Apr 5]. (Wiley Series in Probability and Statistics). Available from: http://doi.wiley.com/10.1002/9780470567333

31. Proust-Lima C, Séne M, Taylor JM, Jacqmin-Gadda H. Joint latent class models for longitudinal and time-to-event data: A review. Stat Methods Med Res. 2014 Feb;23(1):74–90.

32. Vincent P, Larochelle H, Lajoie I, Bengio Y, Manzagol P-A. Stacked Denoising Autoencoders: Learning Useful Representations in a Deep Network with a Local Denoising Criterion. J Mach Learn Res. 2010 Dec;11:3371–408.

33. Zeng C, Zhang W, Doherty M, Persson MSM, Mallen C, Swain S, et al. Initial analgesic prescriptions for osteoarthritis in the United Kingdom, 2000–2016. Rheumatology. 2021 Jan 5;60(1):147–59.

34. Hippisley-Cox J, Coupland C. Unintended effects of statins in men and women in England and Wales: population based cohort study using the QResearch database. BMJ. 2010 May 20;340(may19 4):c2197–c2197.

35. Sarmanova A, Doherty M, Kuo C, Wei J, Abhishek A, Mallen C, et al. Statin use and risk of joint replacement due to osteoarthritis and rheumatoid arthritis: a propensity-score matched longitudinal cohort study. Rheumatology. 2020 Oct 1;59(10):2898–907.

36. Kuo C-F, Grainge MJ, Mallen C, Zhang W, Doherty M. Effect of allopurinol on all-cause mortality in adults with incident gout: propensity score–matched landmark analysis. Rheumatology. 2015 Jul 12;kev246.

37. Royston P, Lambert PC. Flexible parametric survival analysis using Stata: beyond the Cox model. College Station, TX: Stata Press; 2011. 347 p.

38. Millard LAC, Davies NM, Timpson NJ, Tilling K, Flach PA, Smith GD. MR-PheWAS: hypothesis prioritization among potential causal effects of body mass index on many outcomes, using Mendelian randomization. Sci Rep [Internet]. 2015 Dec [cited 2020 Jan 26];5(1). Available from: http://www.nature.com/articles/srep16645

39. Davies NM, Holmes MV, Davey Smith G. Reading Mendelian randomisation studies: a guide, glossary, and checklist for clinicians. BMJ. 2018 Jul 12;k601.

40. Li X, Meng X, Spiliopoulou A, Timofeeva M, Wei W-Q, Gifford A, et al. MR-PheWAS: exploring the causal effect of SUA level on multiple disease outcomes by using genetic instruments in UK Biobank. Ann Rheum Dis. 2018 Jul;77(7):1039–47.

41. Benjamini Y, Yekutieli D. The Control of the False Discovery Rate in Multiple Testing under Dependency. Ann Stat. 2001;29(4):1165–88.

42. Burgess S, Butterworth A, Thompson SG. Mendelian Randomization Analysis With Multiple Genetic Variants Using Summarized Data: Mendelian Randomization Using Summarized Data. Genet Epidemiol. 2013 Nov;37(7):658–65.

43. Bowden J, Davey Smith G, Haycock PC, Burgess S. Consistent Estimation in Mendelian Randomization with Some Invalid Instruments Using a Weighted Median Estimator. Genet Epidemiol. 2016 May;40(4):304–14.

For peer review only

For peer review only - http://bmjopen.bmj.com/site/about/guidelines.xhtml

44. Zhu Z, Zhang F, Hu H, Bakshi A, Robinson MR, Powell JE, et al. Integration of summary data from GWAS and eQTL studies predicts complex trait gene targets. Nat Genet. 2016 May;48(5):481–7.

45. Higgins JPT, Thompson SG. Quantifying heterogeneity in a meta-analysis. Stat Med. 2002 Jun 15;21(11):1539–58.

46. Stewart LA, Tierney JF. To IPD or not to IPD? Advantages and disadvantages of systematic reviews using individual patient data. Eval Health Prof. 2002 Mar;25(1):76–97.

47. Vetrano DL, Roso-Llorach A, Fernández S, Guisado-Clavero M, Violán C, Onder G, et al. Twelve-year clinical trajectories of multimorbidity in a population of older adults. Nat Commun [Internet]. 2020 Dec [cited 2020 Aug 12];11(1). Available from: http://www.nature.com/articles/s41467-020-16780-x

For peer review only

For peer review only - http://bmjopen.bmj.com/site/about/guidelines.xhtml

Figure 1. Overview of the study design and statistical analysis plan for the Comorbidities in Osteoarthritis (CoMO) study.

	Objective 1	Objective 2	Objective 3	Objective 4
Study Design	Case-control and Cohort	Clustering and longitudinal	Cohort	Genomic association
Exposure	OA	OA	Analgesics (NSAIDS, Opioids, paracetamol)	OA
Outcome	Comorbidities	Clusters of comorbidities	Comorbidities	Comorbidities
Statistical methods	Conditional logistic regression,<br>Cox regression	Latent class analysis,<br>Latent class growth analysis<br>Joint latent class analysis	Cox regression<br>Time varying analysis<br>Flexible parametric method	Mendelian randomisation
Reported Outcome	Odds Ratio and Hazard Ratio	Clusters and groups	Hazard Ratio	Coefficients
Participating centres	ALL	ALL	ALL	UK and Netherlands

Objective 5 — **Meta Analysis**

OA- osteoarthritis; NSAIDs- Nonsteroidal anti-inflammatory drugs

For peer review only

For peer review only - http://bmjopen.bmj.com/site/about/guidelines.xhtml

Table 1. Characteristics of the included databases

	Netherlands	Spain	Sweden	UK
Objectives 1-3				
Name of the database	Integrated Primary Care Information (IPCI)	The Spanish Sistema information del Deveolpment de l'Investigació a Atenció Primària (SIDIAP)	Skåne Healthcare Register	Clinical Practice Research Datalink (CPRD)
Settings	Routinely collected primary care database	Routinely collected primary care data	Swedish healthcare in Skane region, primary, specialist and in-patient care	Routinely collected primary care database with linkage database
Size and Coverage	2.2 million (Randomly distributed over the country)	6.5 million (> 85% of total Catalan region)	1.3 million (all residents of the Skane region)	17 million (country-wide, nearly 740 practices)
Start year	1998- (Better coding after 2000)	2006	1998	1993-
Age group	All	All	All	All
Gender	All	All	All	All
Coding system	ICPC	ICD 10	ICD 10	Read codes and ICD 10
Drug prescribed by	GP	GP	GP	GP
Death record (Either date of death and/or cause)	Both date and cause	Only date	Both date and cause (until year 2015)	Only date
Covariates/ additional variables	NA	BMI, Smoking, Alcohol, Social class, cholesterol, and other biomarkers	Education, Income, profession, and sick leave, residential area, region of birth	BMI, Smoking, Alcohol, Deprivation index, Ethnic group

BMI- Body mass index; GP- general practitioners; ICD- International classification of diseases; ICPC- International classification of primary care; NA- Not available

For peer review only

For peer review only - http://bmjopen.bmj.com/site/about/guidelines.xhtml

Table 2. Database for the mendelian randomisation

Name of the database	Rotterdam Cohort Study	UK Biobank
Population coverage	15000	500,000
Age group	>=40 years	40-69 years
Start year -till now	1989-onwards	2010-
Types of data	Radiographic data, joint pain, joint stiffness, of hip, knee, and hand, GWAS, biochemical markers	Genetic and phenotypes

GWAS- Genome Wide Association Studies

For peer review only

For peer review only - http://bmjopen.bmj.com/site/about/guidelines.xhtml

Table 3. List of chronic conditions across four databases

Sl no	Conditions	ICD 10	ICPC	Read Code
1	Anaemia (All types)	D50-D64	B78, B80, B81, B82	D00…, D01…
2	Ankylosing spondylitis	M45.9	NA	N10…
3	Anxiety disorder	F41.0-F41.9	P74, P74.01, P74.02	E200… , Eu41…
4	Asthma	J450-J45.9, J46.9	R96…	66Y…, H33…
5	Benign prostatic hypertrophy (BPH)	N40.9	Y85	K20…, K21…, K22…
6	Cardiac arrhythmias (Atrial Fibrillation)	I47.0-I49.9	K78	Gyu… , G573…
7	Cataract	H25.0-H25.9, H26.1-H26.9	F92	F46… ,
8	Chronic Back pain	M47-M48, M51-M54, M99, G54.4	L02, L03, L86	N12…, N14…,
9	Chronic kidney disease (any cause)	N02.0-N8.8, N11.0-N11.9, N12.9, N15.0-N18.9, N19.9	U99.01	1Z1… , K01…, K02…
10	Chronic neck pain	M54.2	L83…	Nyu… , N11…, N12… , N14…
11	Chronic sinusitis	J32 …	R75…	H13…
12	Chronic obstructive pulmonary diseases	J41.0-J41.8, J42.9, J43.0-J43.9, J44.0-J44.9	R91, R95	H3…
13	Coronary Heart Disease (Including Acute Myocardial infarction, Valvular disease, Angina),	I20.0-I25.0, I34.0-I37.0	K74-K76	G11…, G30…, G31… - G38..
14	Dementia	F00.0-F00.9, F01.0-F03.9, G30.0-G30.9, G31.0-G31.9	P70-P70.02	E00…, Eu0…, F11…
15	Depression	F32.0-F33.9	P76	Eu… , E11…
16	Diabetes mellitus	E10.0-E14.9	T90…, F83.01	C10…, F32…,
17	Dyslipidaemia (Hyper)	E78.1	T93	C32…
18	Eating disorders (Both)			Eu5… , R03…
19	Eczema/ Skin disease	L20.0-L22.9, L26.9	S74, S87, S88	M11…
20	Epilepsy	G40.0-G41.9	N88	F25…
21	Fatigue	F48.0	A04.11	F286…
22	Fibromyalgia	M79.7	L18.01	N248…, N239…
23	Gall bladder stone	K80.0-K80.8	D98-D98.03	781…, J65… , 4G2…,
24	GERD (Gastritis, Oesophageal bleeding, duodenitis, peptic ulcer)	K21 …	D840…	J12… , J13…, J15…
25	Gastrointestinal bleeding	K25.0-K28.9	D84-D87	J11… ,
26	Gout	M10.0-M10.9	T92	C34… , N023…
27	Hearing impairment (All types)	H90.0-H91.9	H83-H86	F59… , ZE87…
28	Heart Failure	150.0-150.9	K77-K77.02	G58…,
29	Hepatitis	K73.0-K73.9	D72-D72.05	J61… , J63…
30	HIV/AIDS	B20-B24	B90	A788… , A789…, AyuC…
31	Hypertension	I10.9, I11.0-I13.9, I15.0-I15.9	K86-K87, F83.02	G20…, G24…, G25…, G26…
32	Hyperthyroidism	E05.0-E05.9	T85	C02…
33	Hypothyroidism	E02.9, E03.0-E03.9	T86	C03…, C04…
34	Inflammatory Bowel Disease (IBD)	K50.0-K52.9	D94-D94.02	J4… ,
35	Irritable Bowel Symptoms (IBS)	K58.1-K58.8		J52…
36	Leukaemia, Lymphoma	C81.0-C86.6, C91.0-C96.9	B72-B73	B60… , B61…, B64…
37	Liver Cirrhosis	K70.0-K71.9, K74.0-K74.6	D97…	J615…
38	Migraine	G43.0-G43.9	N89	F26… ,
39	Multiple sclerosis	G35.9	N86	F20…,
40	Osteoarthritis	M16.0-M16.9, M17.0-M17.9	L89-L91	N05… ,
41	Osteoporosis	M80.0-M82.9	L95…	N33… ,

For peer review only - http://bmjopen.bmj.com/site/about/guidelines.xhtml

42	Other blood vessel disease (Raynaud's disease, Burger's disease)	I73.0-I73.9	K92…	G73… ,
43	Parkinson's disease	G20.9	N87-N87.01	F12…,
44	Peripheral vascular disease (Atherosclerosis)	170.0-170.9	K91	G70…, G71… , G72…
45	Polymyalgia	M35.3	L99.12	N20.11
46	Psoriasis	L40.0-L41.9	S91	M161…
47	Psoriatic arthritis	M07.0-M07.3	L99.13	M160…
48	Rheumatoid Arthritis	M05.0-M05.9	L88… , K71…	N04….
49	Renal stones	N20.0 ….	U95	4G4… , 7B07… , KB12…
50	Schizophrenia and/or psychosis	F20.0-F20.9, F25.0- F25.9	P72	E10…
51	Severe allergy			H17… , SN5…
52	Sjögren's syndrome	M35.0	NA	N002…
53	Systemic Lupus Erythematosus	M32.0, M32.1, M32.8, M32.9	NA	N000…
54	Sleep disorder (Insomnia)	F51.0	P06	Fy0… , 1B1B…
55	Solid malignancy	C00.0-C80.9, D00.0-D09.9, C97.9	A29… , A79, B74 – Y78	B0… - B67… , Byu…
56	Stroke	G45.0-G46.8, I60.0-I63.9, I65.0-166.9, I69.0-I69.4	K89-K90.02	G60… - G68…, F22…
57	Substance abuse/ Drug addiction	F10.0-F19.9	P18, P19…	E24…, Eu1…
58	Thrombotic diseases	I74.0-I74.9	K93, K94…, W99.03	G80… , G81… , G74…
59	Tuberculosis	A15.0-A16.9, B90.9	A70, R70	A1…, A11…
60	Vertigo	H81.4 …	N17-N17.02, H82-H82.03	R004… , F561…
61	Vision problem (Glaucoma and other)	H27.0-H27.9, H40.0-H40.9, H42.0-H42.8	F93…, F94	F45…, F49… ,

ICD- International Classification of Diseases; ICPC- International classification in primary care. All the codes are the primary code initials used in the database.

For peer review only - http://bmjopen.bmj.com/site/about/guidelines.xhtml

Table 4. Group of conditions/Outcome

Group	Conditions
Cardiovascular	Cardiac arrhythmias, Coronary Heart Disease (including AMI, valvular disease, angina), Heart failure, Hypertension, Peripheral vascular disease (claudication, Raynaud syndrome, Buerger's disease), Other blood vessel disease (atherosclerosis and aneurysm), Thrombotic diseases
Gastrointestinal	GERD (Esophageal diseases, gastritis, duodenitis), GI bleeding, Inflammatory bowel disease (IBD), Irritable bowel syndrome (IBS)
Musculoskeletal	Ankylosing spondylitis, Chronic Back pain, Chronic Neck pain, Fibromyalgia, Polymyalgia, Gout, Osteoporosis, Psoriatic arthritis, Rheumatoid arthritis, Sjögren's syndrome, Systemic lupus erythematosus (SLE)
Endocrine	Diabetes mellitus, Dyslipidemia (hyper), Hyperthyroidism, Hypothyroidism
Neurological	Dementia, Epilepsy, Fatigue, Migraine, Multiple sclerosis, Parkinson disease, Stroke
Psychological	Anxiety, Depression, Eating disorders (Anorexia / Bulimia nervosa), Schizophrenia, Sleep disorder (insomnia),
Kidney disease	Chronic kidney disease (any cause), Renal stones
Liver diseases	Gall bladder stone, Hepatitis, Liver cirrhosis
Respiratory	Asthma, Chronic obstructive pulmonary disease (COPD)
Cancer	Leukemia, Lymphoma, Solid Malignancy (any type)
Others	Anemia (all types), Benign prostate hypertrophy (BPH), Cataract, Chronic sinusitis, Eczema/Skin disease, Hearing impairment (all types), Psoriasis, Severe allergy (anaphylactic shock), angioneurotic oedema

For peer review only

For peer review only - http://bmjopen.bmj.com/site/about/guidelines.xhtml

BMJ Open

Comorbidities in Osteoarthritis (CoMO): a multinational study in four European countries

Journal:	*BMJ Open*
Manuscript ID	bmjopen-2021-052816.R1
Article Type:	Protocol
Date Submitted by the Author:	10-Jan-2022
Complete List of Authors:	Swain, Subhashisa; University of Nottingham, Academic Rheumatology<br>Kamps, Anne; Erasmus Medical Center Department of Reumatology, Department of General Practice<br>Runhaar, Jos; Erasmus Medical Center Department of Reumatology, Department of General Practice<br>Dell'Isola, Andrea; Lunds University Faculty of Medicine,<br>Turkiewicz, Aleksandra; Lund University Faculty of Medicine, Dept of Orthopedics<br>Robinson, Danielle ; University of Oxford Nuffield Department of Orthopaedics Rheumatology and Musculoskeletal Sciences,<br>Strauss, V; University of Oxford Nuffield Department of Orthopaedics Rheumatology and Musculoskeletal Sciences,<br>Mallen, Christian; Keele University, Arthritis Research UK Primary Care Centre<br>Kuo, Chang-Fu; Chang Gung Memorial Hospital Linkou Branch,<br>Coupland, Carol; University of Nottingham, Division of Primary Care<br>Doherty, Michael ; University of Nottingham School of Medicine, Academic Rheumatology<br>Sarmanova, Aliya ; University of Bristol,<br>Prieto-Alhambra, Daniel; University of Oxford, Centre for Statistics in Medicine, NDORMS<br>Englund, Martin; Lund University, Dept of Orthopedics<br>Bierma-Zeinstra, Sita; Erasmus University Medical Centre, Department of General Practice<br>Zhang, Weiya; University of Nottingham School of Medicine, Academic Rheumatology
**Primary Subject Heading**:	Rheumatology
Secondary Subject Heading:	Epidemiology, Health services research, Public health, Pharmacology and therapeutics, Rheumatology
Keywords:	EPIDEMIOLOGY, Musculoskeletal disorders < ORTHOPAEDIC & TRAUMA SURGERY, RHEUMATOLOGY

Title- Comorbidities in Osteoarthritis (CoMO): a multinational study in four European countries

Authors

Subhashisa Swain[1,10], Anne Kamps[2], Jos Runhaar[2], Andrea Dell 'Isola[3], Aleksandra Turkiewicz[3], Danielle Robinson[4], Victoria Y Strauss[4], Christian Mallen[5], Chang Fu Kuo[1,6], Carol Coupland[7], Michael Doherty[1,10], Aliya Sarmanova[8], Daniel Prieto Alhambra[4], Martin Englund[3], Sita Bierma-Zeinstra[9], Weiya Zhang[1,10]

1. Academic Rheumatology, School of Medicine, University of Nottingham, UK
2. Department of General Practice, Erasmus MC University Medical Center Rotterdam, The Netherlands
3. Clinical Epidemiology Unit, Orthopaedics, Department of Clinical Sciences Lund, Lund University, Sweden
4. Centre for Statistics in Medicine, NDORMS, University of Oxford, UK
5. School of Medicine, Keele University, UK
6. Division of Rheumatology, Allergy and Immunology, Chang Gung Memorial Hospital, Taiwan.
7. Division of Primary Care, School of Medicine, University of Nottingham, UK
8. Musculoskeletal Research Unit, Bristol Medical School, Translational Health Sciences, University of Bristol, UK
9. Department of General Practice, Department of Orthopaedic Surgery & Sports Medicine, Erasmus MC University Medical Center Rotterdam, The Netherlands
10. Pain Centre Versus Arthritis, University of Nottingham, UK

Corresponding Author:

Professor Weiya Zhang

Academic Rheumatology, School of Medicine

University of Nottingham

Clinical Sciences Building, Nottingham city Hospital

NG5 1PB, Nottingham, UK

Email: weiya.zhang@nottingham.ac.uk

For peer review only - http://bmjopen.bmj.com/site/about/guidelines.xhtml

Abstract

Introduction

Osteoarthritis (OA) is one of the leading chronic conditions in the older population. People with OA are more likely to have one or more other chronic conditions than those without. However, the temporal associations, clusters of the comorbidities, role of analgesics and the causality and variation between populations are yet to be investigated. This paper describes the protocol of a multinational study in four European countries (UK, Netherlands, Sweden, and Spain) exploring comorbidities in people with OA .

Methods and analysis

This multinational study will investigate i) the temporal associations of 61 identified comorbidities with OA, ii) the clusters and trajectories of comorbidities in people with OA, iii) the role of analgesics on incidence of comorbidities in people with OA, iv) the potential biomarkers and causality between OA and the comorbidities, and v) variations between countries.

A combined case-control and cohort study will be conducted to find the temporal association of OA with the comorbidities using the national or regional health databases. Latent class analysis will be performed to identify the clusters at baseline and joint latent class analysis will be used to examine trajectories during the follow-up. A cohort study will be undertaken to evaluate the role of non-steroidal anti-inflammatory drugs (NSAIDs), opioids, and paracetamol on the incidence of comorbidities. Mendelian randomisation will be performed to investigate the potential biomarkers for causality between OA and the comorbidities using the UK Biobank and the Rotterdam Study databases. Finally, a meta-analyses will be used to examine the variations and pool the results from different countries.

Ethics and Dissemination

Research ethics was obtained according to each database requirement. Results will be disseminated through the FOREUM website, scientific meetings, publications, and in partnership with patient organisations.

Key words: Osteoarthritis; Comorbidity; Multimorbidity; Primary care; Analgesics, Mendelian Randomisation, PheWAS

For peer review only - http://bmjopen.bmj.com/site/about/guidelines.xhtml

Strengths and limitations of this study

- This is first ever multicenter study on comorbidities in osteoarthritis in Europe involving nearly 27 million electronic health records
- More than 60 chronic conditions are being studied – representing a wider coverage of diseases
- We will examine the causal association using Mendelian Randomization -PheWAS methods with genetic data collected from two countries
- Same protocol with robust statistical methods will be used across all countries to replicate the findings and examine the variations.
- Possible biases may be introduced by the nature of electronic health records and length of data availability.

For peer review only - http://bmjopen.bmj.com/site/about/guidelines.xhtml

Background

Osteoarthritis (OA) affects 27% of people aged over 45 years at peripheral synovial joints such as knees, hips, hands and feet (1). It is by far the most common form of arthritis, and a leading cause of chronic joint pain and disability in older people (2,3). It is anticipated that the burden of OA will continue to rise in the coming decades because of population ageing and the increasing obesity prevalence – two major risk factors for OA (4,5). Co-occurrence of multiple chronic conditions in an individual with ageing is becoming a norm and OA is not an exception to this.

A recent systematic review has confirmed that people with OA are more likely to have other diseases, especially stroke, peptic ulcer, hypertension, and depression (6). Vast majority of these studies focused on additional presence (comorbidity) of cardiovascular and musculoskeletal conditions only (7–9). Whether these comorbidities just co-exist with OA, share common risk factors with OA, or are causes or consequences of OA remains largely unknown. There was also reporting of wide heterogeneity in definitions of OA and other chronic conditions, diagnosis and recording of diseases, sample sizes, and number of diseases studied in previously published studies included in the review (6). This diversity made the comparison and pooled estimation of comorbidity prevalence difficult.

Comorbidity in OA can occur due to multiple factors. Various hypotheses have been used to explain the existence of comorbidities in general, the most accepted of which are the concordant (diseases sharing similar pathophysiological risk factors) and discordant (diseases not sharing similar pathophysiological risk factors) theories (10). Additionally, prescription of drugs is also reported to be associated with comorbidity and multimorbidity (11). Especially in people with OA, the prescription of analgesics is common, and is associated with increased risk of other conditions such as cardiovascular, gastrointestinal and chronic renal diseases (12,13). Also, having multiple chronic conditions increases the chances of polypharmacy which further escalates the risk of other conditions.

OA is one of the leading conditions reported in multimorbidity research. Exploring the association of OA with other diseases would help in further explaining the burden and pattern of the comorbidity (14). However, the major issue in OA comorbidity research is the low number and specific types of conditions studied (15). Therefore, it is important to develop a consensus on both the count and typology of conditions to be studied to enable comparisons across populations and to derive pooled estimates as appropriate. Further, using uniform

For peer review only - http://bmjopen.bmj.com/site/about/guidelines.xhtml

methods and definitions of diseases in computing these estimates would reduce heterogeneity and make the comparison more reliable.

Understanding the temporal association with comorbidity and disease trajectory is crucial for any chronic condition, and this is possible through studies using longitudinal databases (16). However, one of the limitations of using observational data is that causal associations are difficult to establish, due to the interference of known and unknown confounders. In this study we have used the more recently developed method 'Mendelian Randomization', that can determine causal estimates through combining the use of genetic data and instrumental variable methods."

The burden of diseases in primary care often depends on the population structure, health infrastructure and individual factors such as income and education. Such factors vary between countries, because of the heterogeneity mentioned above there are no robust data comparing OA and its comorbidities between countries. Therefore, the aims of this study were to explore the burden, pattern, and causal factors of comorbidities in people with OA across four European countries using national registration databases in the UK, the Netherlands, Sweden, and Spain.

Objectives

1. To estimate the prevalence, incidence, and time sequence of comorbidities in OA
2. To examine the clusters of comorbidities and trajectories of clusters in OA and associations with death
3. To investigate the associations between commonly used OA drugs, such as non-steroidal anti-inflammatory (NSAIDs) and opioids, and risk of comorbidities
4. To identify the potential biomarkers and causal pathways between OA and the comorbidities
5. To examine the variations of OA comorbidities and clusters across countries.

Methods

Databases

Four routinely collected national (the UK and the Netherlands) or regional (Sweden and Spain) health databases will be used for objectives 1-3. In addition, for objective 4, genomic associations of OA with comorbidities will be examined using two cohort studies from the UK (UK Biobank) (17) and the Netherlands (Rotterdam study) (18). The four national representative and regional databases contain information about the population with primary

For peer review only - http://bmjopen.bmj.com/site/about/guidelines.xhtml

care consultations in four different countries. The longitudinal databases provide information about the diagnosis of the diseases by the general practitioners and some diagnoses made in secondary care, prescription of drugs, deaths, and other health utilisation indicators. The details of the databases and their properties are given in Table 1 and 2.

Participants

People registered with the respective databases aged 18 year or above are eligible for the study.

Patient public involvement (PPI): Three PPI representatives (with OA and all with multiple chronic conditions) were involved in this study through group meetings. Difficulties of living with multiple conditions, lack of research in causal relationship and identification of diseases to be studied and the role of drugs in comorbidity were discussed. They are constantly in touch through providing their inputs at each step of the study.

Definition of Osteoarthritis

OA will be defined as having at least one recorded physician diagnosis of OA for hip, knee, ankle/foot, wrist/hand, or site recorded as 'unspecified' during the study period for the respective database. People with any previous recording of the OA prior to the start date of the study will be excluded.

Comorbidities

We defined comorbidity as the recording of diagnosis of predefined chronic conditions in individuals using either ICD-10 or Read or international classification of primary care (ICPC) code. An extensive list of 61 chronic conditions was prepared from the Quality Outcome Framework (QOF) (19), list of the US Department of Health and Human Services Initiative on Multiple Chronic Conditions (20), global burden of diseases (21) and the Charlson comorbidity index (22). The list has been updated with findings from our systematic review (6) and a previous UK community-based knee pain study (6,23) by including common and important morbidities not included in the above (24,25). A code mapping exercise was conducted to finalise the list of conditions available for all the research centres. The comparison of codes was made, and it was reviewed by four researchers including a clinician from the team. The detailed list of the conditions is given in Table 3.

Covariates

Age and sex will be used in all centres as covariates to adjust in regression models. Additionally, information on body mass index (BMI), smoking, alcohol use, socioeconomic variables such as education level, income, place of birth (to identify those who immigrated to

For peer review only - http://bmjopen.bmj.com/site/about/guidelines.xhtml

the country), and residential area, marriage (or registered partner) will be included when available. For calculating severity of the comorbidities in an individual, Elixhauser comorbidity index will be used to estimate the impact of comorbidities on death (26,27). Missing data on covariates will be substituted using multiple imputation methods, provided that the data is missing at random, if applicable.

Data Harmonisation:

Firstly, we carried out a code mapping exercise for identification of people with osteoarthritis (OA) and other comorbidities. We developed a list of chronic conditions and each centres shared the list of codes to be used for the conditions, such as Read code in CPRD (UK), ICPC2 (Rotterdam) and ICD-10 for Lund and Spanish as per their database. The code lists were compared and edited to maintain the uniformity. The list was screened by verified by two researchers and two GPs. We also used unform definition for inclusion of condition e.g. at least one recording of the chronic conditions. Because all the centres did not have all the listed comorbidities, a minimum number of chronic conditions and covariates common in all the database were identified to be studied. Similarly, we decided to have a minimum follow-up study of 5 years and centres with more registration period can use the entire length of data available.

Study design and data analysis

Summary of the study design and analysis is provided in Figure 1. All the centres plan to use same statistical analysis plan to investigate each objective.

Ethics and Dissemination:

The study has obtained the following ethics approvals: UK- Independent Scientific Advisory Council (ISAC) 19/30R, The Netherlands- The Integrated Primary Care Information (IPCI) registration no. 11/2019, Spain - the Information System for the Development of Research in Primary Care (SIDIAP), 4R19/011 , Sweden –Ethical Review Authority, Skåne Healthcare Register, ‘Dnr 2011-432, Dnr 2014-276, and Dnr 2018-233. The registry databases are made available anonymised for the research purposes.

Objective 1 – Prevalence, incidence, and time sequence of comorbidities in OA

A combined retrospective and prospective study of OA cases and sex, age (+/-2 years), first year of registration, and practice matched controls (1:1-4) without OA (28) will be used to determine the prevalence, incidence, and time sequence of comorbidities in OA. Incident OA

For peer review only - http://bmjopen.bmj.com/site/about/guidelines.xhtml

cases will be identified, and the first diagnosis date will be used as the starting point (index date). For controls the same index date as their matched case will be used. They will be both retrospectively reviewed for prior diagnoses of comorbidities and prospectively followed-up for posterior new comorbidities. In the retrospective analysis the prevalence and 95% confidence interval (CI) of each specific comorbidity will be calculated separately in OA cases and matched controls using the number of people diagnosed with the comorbidity divided by the total number of OA cases or controls at the index date. The prevalence of each comorbidity in OA cases and matched controls will be calculated for given time intervals prior to the index date of 0-1, 0-5 and 0-10 years separately to assess observational bias (28). Discrete time intervals of 1-5, and 5-10 years before will also be used to estimate the prevalence to minimise consultation bias/misclassification bias of OA (if possible). Logistic regression will be used to calculate the odds ratios (OR) for each comorbidity unadjusted and adjusted for BMI, smoking and alcohol consumption.

For the prospective analysis participants with incident OA but without the specific comorbidity of interest at the index date (i.e., people at risk) and matched controls without OA will be followed up until the date of the first diagnosis of the comorbidity, deregistration, or death whichever comes first. The cumulative incidence will be calculated for each comorbidity in OA cases and matched controls at 1, 3, 5, 10, 15, 20 years (based on the data available) after the index date to examine the dynamic change of developing comorbidities during follow-up. Kaplan-Meier survival curves will be used to display the cumulative probability in OA and non-OA groups. Proportional hazard assumption will be tested using Schoenfeld residual plots. The Cox regression model will be used to calculate hazard ratios (HR) for each comorbidity unadjusted and adjusted for age, sex, practice, BMI, smoking and alcohol consumption. This hybrid design has been previously used by us to examine the temporality of associations between other rheumatic musculoskeletal diseases (RMDs) (e.g., gout and lupus) and comorbidities (28,29).

Objective 2 – Clusters and impact of comorbidities in people with OA

For each dataset, an 80%: 20% split into the training and testing data will be introduced. The following analysis in objective 2 will be first employed into the training dataset and then tested its generalisability in the testing dataset. At baseline, clusters of people based on 61 comorbidities will be identified using Latent class (i.e., Gaussian mixture models algorithms of cluster) analysis (30). For each model, we will examine the association between clusters and covariates using multinomial logistic regressions. The distinctness of clusters will be

For peer review only - http://bmjopen.bmj.com/site/about/guidelines.xhtml

examined by comparing covariates among clusters. The optimal model is the one where most clusters found in the training data are also identified independently in the testing data and clusters have most distinct patients' characteristics. We will then use both latent trajectory analysis, such as joint latent class models (31), and unsupervised machine learning approach, such as deep autoencoder or recurrent neural networks (32), to identify distinct clusters of new comorbidity numbers development over time and their association with mortality with adjustment for baseline covariates.

Objective 3 - Association between analgesics and incident comorbidities

A cohort study will be undertaken for this objective to evaluate the contribution of common analgesics for OA to the development of comorbidity such as NSAIDs, opioids and paracetamol. We are interested in the interaction between OA and use of drugs on the incidence of comorbidities, i.e. to evaluate if the drug use in persons with OA poses increased or decreased risk of comorbidities compared to persons without OA and/or analgesics. Individual comorbidity, as well as clusters of comorbidities identified from Objective 2 will be examined as outcomes. The 61 comorbidities in our study will be further categorised into eight groups, specifically: musculoskeletal (MSK), respiratory, neurodegenerative, psychological/psychiatric, cancer, cardiovascular, metabolic, renal problem, liver diseases, gastrointestinal (GI) and others. (Table 4) The prospective cohort established from Objective 1 will form the source population for this objective. Individuals with incident OA will be identified from the database and the first diagnosis date will be used as index date for follow-up. Individuals without OA during the study period will be selected and matched with cases by age, sex, and practice. The same index date will be given from their matched OA cases. Individuals with analgesics prescriptions prior to the index date will be excluded (or recorded as a confounding factor to be adjusted as appropriate). Only analgesic prescriptions after the index date will be considered for this analysis. Prescriptions will be quantified as number of prescriptions within year 1 (initial use, primary analysis) (33), 2, 3, 4, 5 etc. It will also be dichotomised as episodic (e.g., at least one gap of ≥90 days between prescriptions) and continuous (no gap of more less than 90 days) users as appropriate (34,35). Analgesic use will be included in the model as a risk factor together with OA diagnosis (yes/no, primary exposure) to examine the independent risk of each variable (OA and analgesics), as well as the interaction between the two to the development of comorbidity. Dose response relationship will be examined using number of prescriptions during the exposure window examined. The effect of stopping analgesics will also be examined by looking into the

For peer review only - http://bmjopen.bmj.com/site/about/guidelines.xhtml

patterns of analgesic prescriptions, e.g., stopping analgesics after initial use in year 1 versus continuous use of analgesics afterwards. For the primary analysis (initial prescriptions within year 1), a landmark analysis will be used to minimise the immortal time bias where the follow up will start after 12 months from the index date (36). Participants at risk (i.e., without a specific comorbidity of interest) at the landmark date will be followed up until the first diagnosis of the comorbidity, deregistration, or death whichever comes first. For secondary analyses, time varying covariate analysis will be used to examine the long-term, episodic/continuous use of analgesics after the index date and interaction between OA and analgesics in the development of the comorbidity. The propensity score matching or the inverse probability weighing methods will be used to adjust for confounding by indication during the follow-up as appropriate. Depending on the country-specific drug use patterns, we may modify this definition to allow for short brakes in between the episodes. Cox-regression model will be used to calculate the HR and 95% CI. We will use flexible parametric models using restricted cubic splines (developed by Lambert, “stpm2” in Stata) to estimate the HRs and differences in time to diagnosis of comorbidities (outcome) with drugs as time-varying to account for non-proportional hazards (37).

Objective 4 – Potential causal pathways between OA and the comorbidities

We will perform a Mendelian Randomisation (MR) phenome-wide association (MR-PheWAS) study(38) to examine the causal relationship between OA, its phenotypes, biomarkers or risk factors and comorbidities using the UK Biobank and the Rotterdam Study database.

We will use the Rotterdam Study and the UK Biobank jointly for this objective. This is because that the Rotterdam Study is an OA cohort with deep phenotypes and biomarkers of OA, whereas the UK Biobank is a primary cohort for cancer and multiple disease outcomes, and both have detailed genetic variants. We will use two sample MR approach, i.e., to establish an association between OA and genetic variants in the Rotterdam Cohort to identify genetic instrumental variables (IV), e.g., a set of single nucleotide polymorphisms (SNPs) associated with OA (or a deep phenotype, biomarker, or risk factor of OA). We will then undertake the MR-PheWAS analysis to examine the causal effects of the OA IV on comorbidities in the UK Biobank. The MR method has been widely used in real world data to examine the causal relationship between IV and specific disease, under two assumptions: [1] genetic variants are randomly assigned in the population; and [2] genetic variants can only be the cause not consequence of disease (39). The PheWAS is a series of case control studies to

For peer review only - http://bmjopen.bmj.com/site/about/guidelines.xhtml

estimate the associations between the IV and multiple disease outcomes (38,40). The combination of the two permits investigation of the causal effects of OA on multiple disease outcomes.

The MR-PheWAS analysis includes three steps. Firstly, we will identify the genetic variants that are associated with OA - IV. Secondly, we will undertake the PheWAS analysis – a series of case control analyses to estimate the associations between the IV and other disease outcomes (38,40) , with an adjustment for multiple testing using the false discovery rate (FDR) methods (41). Thirdly, we will implement conventional MR analysis to investigate the causal effects of the OA IV on comorbidities (39). An inverse variance weighted (IVW) method will be used to pool the associations (ORs) as appropriate (42). The MR-Egger regression analysis will be used to count for the pleiotropic effect - the effects of one genetic variant on multiple outcomes (43). The heterogeneity in dependent instruments (HEIDI) test will be used to exclude the cross-phenotype associations caused by genetic linkage (44).

With the MR-PheWAS study, the OR can be interpreted as causal association. We are primarily interested in the causality from OA to comorbidities. We are also interested in inflammatory (e.g., CRP), metabolic (e.g., gut microbiome) and biomechanics (e.g., BMI) biomarkers and deep phenotypes of OA such as knee, hip, and hand OA with and without symptoms. This will be undertaken if it is feasible within 3 years of this funded project, otherwise will be considered as our future research agenda.

Objective 5 – Variation of OA comorbidity patterns across countries

We will use meta-analyses (MA) to examine the variation between countries and to pool the data as appropriate. Estimates from first three objectives such as prevalence, incidence, OR, HR and 95% CI for each specific comorbidity across different populations will be distributed in a forest plot. Heterogeneity will be examined using the I^2 statistic and the Q test (45). Results will be pooled if they are homogenous based on the I^2 value using the fixed effects model, otherwise the reasons for the heterogeneity will be investigated. Random effects models will be used to pool the results if the reasons for the heterogeneity cannot be identified and if the overall pooling is appropriate. Individual patient data (IPD) meta-analysis may be used to help identify the reasons for heterogeneity (46). Common clusters and trajectories as well as burdens of comorbidities will also be compared between populations.

Feasibility and sample size

For peer review only - http://bmjopen.bmj.com/site/about/guidelines.xhtml

To detect minimum incidence of 1% comorbidity (required for cluster analysis) with a minimum clinical important difference of hazard ratio (HR)1.2, and 90% power of the study, the estimated sample size was 197561 for 1581 events. It was calculated using STATA, with a correlation= 0.2, standard deviation of 0.5, proportion of withdrawal= 0.20, alpha=0.05. The initial check with the registry database revealed to have minimum required sample size for the study.

Discussion

This study will be the largest epidemiological study on comorbidities of OA in primary care. One of the key advantages of this multinational study is the use of the same protocol to measure the burden of comorbidities in primary care settings in four European countries to ensure reproducibility and comparison. There is scant evidence on the comorbidities in people with OA, and this approach should help to identify the leading and most important associations before and after presenting clinical OA (the index date). Further advantages of this study are the large and representative populations studied and the same/similar extensive list of chronic conditions for identifying comorbidity clusters. Often comorbidities accumulate with age over time and the large primary care databases in this study have the advantage of having long follow-up time which will enable us to detect the incidence of comorbidities. Also, longer follow up would help to identify the picture of the trajectory of the diseases (47). Both the incidence and the trajectories of comorbidity clusters are highlighted as key elements needed in current research in multimorbidity, so findings from this study should help to fill the knowledge gaps on multimorbidity in OA.

The relationship between chronic conditions and polypharmacy is a complex area of research. The count of the medications and more importantly the nature of prescribed drugs may be responsible for developing many new comorbidities in people with OA. We aim to explore the associations of the most commonly prescribed drugs in OA, such as NSAIDs, with the incidence of a wide range of comorbidities, which will be the first time that conditions other than established comorbidities such as psychological conditions and endocrine diseases will be examined. Finally, the causality study will further explore the associations at genetic levels and phenotypes, which will be novel in OA research. Using a two sample MR approach - one for OA deep phenotypes and the other for other chronic conditions maximises the potentials of sample size, disease phenotypes and comorbidity spectrum to better explore the causal pathways between OA and comorbidity.

For peer review only - http://bmjopen.bmj.com/site/about/guidelines.xhtml

There are some limitations to this study. Firstly, there are inherent issues in the nature of electronic health records with respect to possible misdiagnosis, ascertainment biases, under- or over-recording, and changes in databases due to change in coding structures. Also, the analysis will be restricted to fewer covariates in some databases due to missing information on lifestyle factors such as physical activities and diet. Even though the databases have different durations of data available, if possible we will use a common follow-up time for objective five. Another important limitation is that we do not have information on quality of life and other outcomes to measure functional limitations recorded in the database. Chronic conditions, especially comorbidities recorded in general practices, depend on multiple factors such as population structure, health care facilities, health policies, and the nature of the national databases. A major strength of this study is that it will include medical records on approximately 27 million people in four European countries. Also, the study will cover the sequence of research questions in comorbidity or multimorbidity starting from the burden through to the causality and variation. Such a research model can be used for other similar multimorbidity studies. The expected results should inform health professionals in primary care settings with respect to management of people with OA and associated comorbidities.

Status of the study

All the centres have obtained the necessary approvals for using the database in 2020. A consensus has been made on the code mapping exercise. The statistical analysis will be explained in detail in each of the publications. The team is expected to produce results by mid-2021.

Acknowledgements: We thank the Patient Research Participants (PRP) members Jenny Cockshull, Stevie Vanhegan, and Irene Pitsillidou for their involvement since the beginning of the project. We would like to thank the FOREUM for financially supporting the research. The authors would like to acknowledge Keele University's Prognosis and Consultation Epidemiology Research Group who have given us permission to utilise the Code Lists (©2014).

Contributor and guarantor information:

WZ, MD, CC, SMA, ME, DA conceived and designed the study. SS, AK, AD, AT, DR, and VS developed the methods and will perform the analysis, and interpretation of the results. CC, WZ, JR, AS, CFK, VS, and AT will supervise the statistical analysis. CM and MD will guide with clinical interpretations of the results. SS drafted this manuscript and all authors contributed to the critical revision of the manuscript for important intellectual content. The corresponding author attests that all listed authors meet authorship criteria and that no others meeting the criteria have been omitted.

For peer review only - http://bmjopen.bmj.com/site/about/guidelines.xhtml

Funding: This work was supported by Foundation for Research in Rheumatology (FOREUM) grant (2019-2022), The Swedish Research Council (2020-01103), Governmental funding of clinical research within the national health services (ALF), and The Swedish Rheumatism Association. CM is funded by the National Institute for Health Research (NIHR) Applied Research Collaboration West Midlands, the National Institute for Health Research (NIHR) School for Primary Care Research and a National Institute for Health Research (NIHR) Research Professorship in General Practice (NIHR-RP-2014-04-026)

Role of the funding sources: The sponsors did not participate in the design and conduct of the study; collection, management, analysis, and interpretation of the data; or preparation, review, or approval of the manuscript and the decision to submit the manuscript for publication.

Competing interests: WZ declares serving as an advisory board for Ely Lilly (Ixekizumad, 2020) and Regeneron (Fasinomab, 2020). ME declares serving as an advisory Panel Board Member for Pfizer (Nov 2019, Tanezumab). CM provided advice to BMS on recruiting to a non-pharmacological atrial fibrillation trial. The other authors report no financial competing interests.

Copy right: The Corresponding Author has the right to grant on behalf of all authors and does grant on behalf of all authors, an exclusive licence (or non-exclusive for government employees) on a worldwide basis to the BMJ Publishing Group Ltd to permit this article (if accepted) to be published in BMJ editions and any other BMJPGL products and sublicences such use and exploit all subsidiary rights, as set out in our licence.

Studies involving humans or animals: No direct participant recruitment was done for the study.

Data sharing statement: We used anonymised data on individual patients on which the analysis, results, and conclusions reported in the paper are based. The used data is not distributable under licence. However, the relevant data can be obtained directly from the respective agencies. The codes developed for the analysis can be available upon a valid request.

Figure legend

Figure 1. Overview of the study design and statistical analysis plan for the Comorbidities in Osteoarthritis (CoMO) study.

For peer review only

Reference

1. Turkiewicz A, Petersson IF, Björk J, Hawker G, Dahlberg LE, Lohmander LS, et al. Current and future impact of osteoarthritis on health care: a population-based study with projections to year 2032. Osteoarthritis Cartilage. 2014 Nov;22(11):1826–32.

2. Badley EM. The effect of osteoarthritis on disability and health care use in Canada. J Rheumatol Suppl. 1995 Feb;43:19–22.

3. McDonough CM, Jette AM. The Contribution of Osteoarthritis to Functional Limitations and Disability. Clin Geriatr Med. 2010 Aug;26(3):387–99.

4. Cieza A, Causey K, Kamenov K, Hanson SW, Chatterji S, Vos T. Global estimates of the need for rehabilitation based on the Global Burden of Disease study 2019: a systematic analysis for the Global Burden of Disease Study 2019. The Lancet. 2020 Dec;396(10267):2006–17.

5. World Health Organization. Musculoskeletal conditions [Internet]. [cited 2021 Apr 6]. Available from: https://www.who.int/news-room/fact-sheets/detail/musculoskeletal-conditions

6. Swain S, Sarmanova A, Coupland C, Doherty M, Zhang W. Comorbidities in Osteoarthritis: A systematic review and meta-analysis of observational studies. 2019 Jun 17 [cited 2019 Jun 30]; Available from: http://doi.wiley.com/10.1002/acr.24008

7. Suri P, Morgenroth DC, Hunter DJ. Epidemiology of osteoarthritis and associated comorbidities. PM R. 2012 May;4(5 Suppl):S10-19.

8. Hall AJ, Stubbs B, Mamas MA, Myint PK, Smith TO. Association between osteoarthritis and cardiovascular disease: Systematic review and meta-analysis. Eur J Prev Cardiol. 2016;23(9):938–46.

9. Stubbs B, Aluko Y, Myint PK, Smith TO. Prevalence of depressive symptoms and anxiety in osteoarthritis: a systematic review and meta-analysis. Age Ageing. 2016 Mar 1;45(2):228–35.

10. Ricci-Cabello I, Stevens S, Kontopantelis E, Dalton ARH, Griffiths RI, Campbell JL, et al. Impact of the Prevalence of Concordant and Discordant Conditions on the Quality of Diabetes Care in Family Practices in England. Ann Fam Med. 2015 Nov 1;13(6):514–22.

11. Calderón-Larrañaga A, Poblador-Plou B, González-Rubio F, Gimeno-Feliu LA, Abad-Díez JM, Prados-Torres A. Multimorbidity, polypharmacy, referrals, and adverse drug events: Are we doing things well? Br J Gen Pract. 2012;62(605):e821–6.

12. Jüni P, Nartey L, Reichenbach S, Sterchi R, Dieppe PA, Egger M. Risk of cardiovascular events and rofecoxib: cumulative meta-analysis. The Lancet. 2004 Dec;364(9450):2021–9.

13. Castellsague J, Riera-Guardia N, Calingaert B, Varas-Lorenzo C, Fourrier-Reglat A, Nicotra F, et al. Individual NSAIDs and Upper Gastrointestinal Complications: A Systematic Review and Meta-Analysis of Observational Studies (the SOS Project). Drug Saf. 2012 Dec;35(12):1127–46.

14. Lowe D.B., Taylor M.J., Hill S.J. Cross-sectional examination of musculoskeletal conditions and multimorbidity: influence of different thresholds and definitions on prevalence and association estimates. BMC Res Notes. 2017;10(1):51.

15. John R, Kerby DS, Hagan Hennessy C. Patterns and Impact of Comorbidity and Multimorbidity Among Community-Resident American Indian Elders. The Gerontologist. 2003 Oct 1;43(5):649–60.

For peer review only - http://bmjopen.bmj.com/site/about/guidelines.xhtml

16. Alaeddini A., Jaramillo C.A., Faruqui S.H.A., Pugh M.J. Mining Major Transitions of Chronic Conditions in Patients with Multiple Chronic Conditions. Methods Inf Med. 2017;56(5):391–400.

17. Sudlow C, Gallacher J, Allen N, Beral V, Burton P, Danesh J, et al. UK Biobank: An Open Access Resource for Identifying the Causes of a Wide Range of Complex Diseases of Middle and Old Age. PLOS Med. 2015 Mar 31;12(3):e1001779.

18. Engelhart MJ, Geerlings MI, Meijer J, Kiliaan A, Ruitenberg A, van Swieten JC, et al. Inflammatory Proteins in Plasma and the Risk of Dementia: The Rotterdam Study. Arch Neurol. 2004 May 1;61(5):668.

19. Quality and Outcome Framework (QOF) [Internet]. [cited 2018 Mar 21]. Available from: https://digital.nhs.uk/article/8910/Quality-and-Outcome-Framework-QOF-Indicators-No-Longer-In-QOF-INLIQ-Enhanced-Services-ES-Vaccinations-and-Immunisations-V-I-and-GMS-Core-Contract-CC-extraction-specifications-business-rules-

20. Medicare C for, Baltimore MS 7500 SB, Usa M. CC_Main [Internet]. 2017 [cited 2018 Oct 24]. Available from: https://www.cms.gov/Research-Statistics-Data-and-Systems/Statistics-Trends-and-Reports/Chronic-Conditions/CC_Main.html

21. James SL, Abate D, Abate KH, Abay SM, Abbafati C, Abbasi N, et al. Global, regional, and national incidence, prevalence, and years lived with disability for 354 diseases and injuries for 195 countries and territories, 1990–2017: a systematic analysis for the Global Burden of Disease Study 2017. The Lancet. 2018 Nov;392(10159):1789–858.

22. Charlson ME, Pompei P, Ales KL, MacKenzie CR. A new method of classifying prognostic comorbidity in longitudinal studies: Development and validation. J Chronic Dis. 1987 Jan 1;40(5):373–83.

23. Sarmanova A, Fernandes GS, Richardson H, Valdes AM, Walsh DA, Zhang W, et al. Contribution of central and peripheral risk factors to prevalence, incidence and progression of knee pain: a community-based cohort study. Osteoarthritis Cartilage. 2018 Nov;26(11):1461–73.

24. Charlson M, Szatrowski TP, Peterson J, Gold J. Validation of a combined comorbidity index. J Clin Epidemiol. 1994 Nov 1;47(11):1245–51.

25. Quan H, Li B, Couris CM, Fushimi K, Graham P, Hider P, et al. Updating and Validating the Charlson Comorbidity Index and Score for Risk Adjustment in Hospital Discharge Abstracts Using Data From 6 Countries. Am J Epidemiol. 2011 Mar 15;173(6):676–82.

26. van Walraven C, Austin PC, Jennings A, Quan H, Forster AJ. A Modification of the Elixhauser Comorbidity Measures Into a Point System for Hospital Death Using Administrative Data: Med Care. 2009 Jun;47(6):626–33.

27. Elixhauser A, Steiner C, Harris DR, Coffey RM. Comorbidity measures for use with administrative data. Med Care. 1998 Jan;36(1):8–27.

28. Kuo C-F, Grainge MJ, Mallen C, Zhang W, Doherty M. Comorbidities in patients with gout prior to and following diagnosis: case-control study. Ann Rheum Dis. 2014 Nov 14;annrheumdis-2014-206410.

29. Swain S, Coupland C, Mallen C, Kuo CF, Sarmanova A, Bierma-Zeinstra SMA, et al. Temporal relationship between osteoarthritis and comorbidities: a combined case control and cohort study in the UK primary care setting. Rheumatology. 2021 Mar 1;keab067.

For peer review only

For peer review only - http://bmjopen.bmj.com/site/about/guidelines.xhtml

30. Collins LM, Lanza ST. Latent Class and Latent Transition Analysis [Internet]. Hoboken, NJ, USA: John Wiley & Sons, Inc.; 2009 [cited 2020 Apr 5]. (Wiley Series in Probability and Statistics). Available from: http://doi.wiley.com/10.1002/9780470567333

31. Proust-Lima C, Séne M, Taylor JM, Jacqmin-Gadda H. Joint latent class models for longitudinal and time-to-event data: A review. Stat Methods Med Res. 2014 Feb;23(1):74–90.

32. Vincent P, Larochelle H, Lajoie I, Bengio Y, Manzagol P-A. Stacked Denoising Autoencoders: Learning Useful Representations in a Deep Network with a Local Denoising Criterion. J Mach Learn Res. 2010 Dec;11:3371–408.

33. Zeng C, Zhang W, Doherty M, Persson MSM, Mallen C, Swain S, et al. Initial analgesic prescriptions for osteoarthritis in the United Kingdom, 2000–2016. Rheumatology. 2021 Jan 5;60(1):147–59.

34. Hippisley-Cox J, Coupland C. Unintended effects of statins in men and women in England and Wales: population based cohort study using the QResearch database. BMJ. 2010 May 20;340(may19 4):c2197–c2197.

35. Sarmanova A, Doherty M, Kuo C, Wei J, Abhishek A, Mallen C, et al. Statin use and risk of joint replacement due to osteoarthritis and rheumatoid arthritis: a propensity-score matched longitudinal cohort study. Rheumatology. 2020 Oct 1;59(10):2898–907.

36. Kuo C-F, Grainge MJ, Mallen C, Zhang W, Doherty M. Effect of allopurinol on all-cause mortality in adults with incident gout: propensity score–matched landmark analysis. Rheumatology. 2015 Jul 12;kev246.

37. Royston P, Lambert PC. Flexible parametric survival analysis using Stata: beyond the Cox model. College Station, TX: Stata Press; 2011. 347 p.

38. Millard LAC, Davies NM, Timpson NJ, Tilling K, Flach PA, Smith GD. MR-PheWAS: hypothesis prioritization among potential causal effects of body mass index on many outcomes, using Mendelian randomization. Sci Rep [Internet]. 2015 Dec [cited 2020 Jan 26];5(1). Available from: http://www.nature.com/articles/srep16645

39. Davies NM, Holmes MV, Davey Smith G. Reading Mendelian randomisation studies: a guide, glossary, and checklist for clinicians. BMJ. 2018 Jul 12;k601.

40. Li X, Meng X, Spiliopoulou A, Timofeeva M, Wei W-Q, Gifford A, et al. MR-PheWAS: exploring the causal effect of SUA level on multiple disease outcomes by using genetic instruments in UK Biobank. Ann Rheum Dis. 2018 Jul;77(7):1039–47.

41. Benjamini Y, Yekutieli D. The Control of the False Discovery Rate in Multiple Testing under Dependency. Ann Stat. 2001;29(4):1165–88.

42. Burgess S, Butterworth A, Thompson SG. Mendelian Randomization Analysis With Multiple Genetic Variants Using Summarized Data: Mendelian Randomization Using Summarized Data. Genet Epidemiol. 2013 Nov;37(7):658–65.

43. Bowden J, Davey Smith G, Haycock PC, Burgess S. Consistent Estimation in Mendelian Randomization with Some Invalid Instruments Using a Weighted Median Estimator. Genet Epidemiol. 2016 May;40(4):304–14.

For peer review only

44. Zhu Z, Zhang F, Hu H, Bakshi A, Robinson MR, Powell JE, et al. Integration of summary data from GWAS and eQTL studies predicts complex trait gene targets. Nat Genet. 2016 May;48(5):481–7.

45. Higgins JPT, Thompson SG. Quantifying heterogeneity in a meta-analysis. Stat Med. 2002 Jun 15;21(11):1539–58.

46. Stewart LA, Tierney JF. To IPD or not to IPD? Advantages and disadvantages of systematic reviews using individual patient data. Eval Health Prof. 2002 Mar;25(1):76–97.

47. Vetrano DL, Roso-Llorach A, Fernández S, Guisado-Clavero M, Violán C, Onder G, et al. Twelve-year clinical trajectories of multimorbidity in a population of older adults. Nat Commun [Internet]. 2020 Dec [cited 2020 Aug 12];11(1). Available from: http://www.nature.com/articles/s41467-020-16780-x

For peer review only

For peer review only - http://bmjopen.bmj.com/site/about/guidelines.xhtml

Table 1. Characteristics of the included databases

	Netherlands	Spain	Sweden	UK
Objectives 1-3				
Name of the database	Integrated Primary Care Information (IPCI)	The Spanish Sistema information del Deveolpment de l'Investigació a Atenció Primària (SIDIAP)	Skåne Healthcare Register	Clinical Practice Research Datalink (CPRD)
Settings	Routinely collected primary care database	Routinely collected primary care data	Swedish healthcare in Skane region, primary, specialist and in-patient care	Routinely collected primary care database with linkage database
Size and Coverage	2.2 million (Randomly distributed over the country)	6.5 million (> 85% of total Catalan region)	1.3 million (all residents of the Skane region)	17 million (country-wide, nearly 740 practices)
Start year	1998- (Better coding after 2000)	2006	1998	1993-
Age group	All	All	All	All
Gender	All	All	All	All
Coding system	ICPC	ICD 10	ICD 10	Read codes and ICD 10
Drug prescribed by	GP	GP	GP	GP
Death record (Either date of death and/or cause)	Both date and cause	Only date	Both date and cause (until year 2015)	Only date
Covariates/ additional variables	NA	BMI, Smoking, Alcohol, Social class, cholesterol, and other biomarkers	Education, Income, profession, and sick leave, residential area, region of birth	BMI, Smoking, Alcohol, Deprivation index, Ethnic group

BMI- Body mass index; GP- general practitioners; ICD- International classification of diseases; ICPC- International classification of primary care; NA- Not available

For peer review only

Table 2. Database for the mendelian randomisation

Name of the database	Rotterdam Cohort Study	UK Biobank
Population coverage	15000	500,000
Age group	>=40 years	40-69 years
Start year -till now	1989-onwards	2010-
Types of data	Radiographic data, joint pain, joint stiffness, of hip, knee, and hand, GWAS, biochemical markers	Genetic and phenotypes

GWAS- Genome Wide Association Studies

For peer review only

For peer review only - http://bmjopen.bmj.com/site/about/guidelines.xhtml

Table 3. List of chronic conditions across four databases

Sl no	Conditions	ICD 10	ICPC	Read Code
1	Anaemia (All types)	D50-D64	B78, B80, B81, B82	D00…, D01…
2	Ankylosing spondylitis	M45.9	NA	N10…
3	Anxiety disorder	F41.0-F41.9	P74, P74.01, P74.02	E200… , Eu41…
4	Asthma	J450-J45.9, J46.9	R96…	66Y…, H33…
5	Benign prostatic hypertrophy (BPH)	N40.9	Y85	K20…, K21…, K22…
6	Cardiac arrhythmias (Atrial Fibrillation)	I47.0-I49.9	K78	Gyu… , G573…
7	Cataract	H25.0-H25.9, H26.1-H26.9	F92	F46… ,
8	Chronic Back pain	M47-M48, M51-M54, M99, G54.4	L02, L03, L86	N12…, N14…,
9	Chronic kidney disease (any cause)	N02.0-N8.8, N11.0-N11.9, N12.9, N15.0-N18.9, N19.9	U99.01	1Z1… , K01…, K02…
10	Chronic neck pain	M54.2	L83…	Nyu… , N11…, N12… , N14…
11	Chronic sinusitis	J32 …	R75…	H13…
12	Chronic obstructive pulmonary diseases	J41.0-J41.8, J42.9, J43.0-J43.9, J44.0-J44.9	R91, R95	H3…
13	Coronary Heart Disease (Including Acute Myocardial infarction, Valvular disease, Angina),	I20.0-I25.0, I34.0-I37.0	K74-K76	G11…, G30…, G31… - G38..
14	Dementia	F00.0-F00.9, F01.0-F03.9, G30.0-G30.9, G31.0-G31.9	P70-P70.02	E00…, Eu0…, F11…
15	Depression	F32.0-F33.9	P76	Eu… , E11…
16	Diabetes mellitus	E10.0-E14.9	T90…, F83.01	C10…, F32…,
17	Dyslipidaemia (Hyper)	E78.1	T93	C32…
18	Eating disorders (Both)			Eu5… , R03…
19	Eczema/ Skin disease	L20.0-L22.9, L26.9	S74, S87, S88	M11…
20	Epilepsy	G40.0-G41.9	N88	F25…
21	Fatigue	F48.0	A04.11	F286…
22	Fibromyalgia	M79.7	L18.01	N248…, N239…
23	Gall bladder stone	K80.0-K80.8	D98-D98.03	781…, J65… , 4G2…,
24	GERD (Gastritis, Oesophageal bleeding, duodenitis, peptic ulcer)	K21 …	D840…	J12… , J13…, J15…
25	Gastrointestinal bleeding	K25.0-K28.9	D84-D87	J11… ,
26	Gout	M10.0-M10.9	T92	C34… , N023…
27	Hearing impairment (All types)	H90.0-H91.9	H83-H86	F59… , ZE87…
28	Heart Failure	150.0-150.9	K77-K77.02	G58…,
29	Hepatitis	K73.0-K73.9	D72-D72.05	J61… , J63…
30	HIV/AIDS	B20-B24	B90	A788… , A789…, AyuC…
31	Hypertension	I10.9, I11.0-I13.9, I15.0-I15.9	K86-K87, F83.02	G20…, G24…, G25…, G26…
32	Hyperthyroidism	E05.0-E05.9	T85	C02…
33	Hypothyroidism	E02.9, E03.0-E03.9	T86	C03…, C04…
34	Inflammatory Bowel Disease (IBD)	K50.0-K52.9	D94-D94.02	J4… ,
35	Irritable Bowel Symptoms (IBS)	K58.1-K58.8		J52…
36	Leukaemia, Lymphoma	C81.0-C86.6, C91.0-C96.9	B72-B73	B60… , B61…, B64…
37	Liver Cirrhosis	K70.0-K71.9, K74.0-K74.6	D97…	J615…
38	Migraine	G43.0-G43.9	N89	F26… ,
39	Multiple sclerosis	G35.9	N86	F20…,
40	Osteoarthritis	M16.0-M16.9, M17.0-M17.9	L89-L91	N05… ,
41	Osteoporosis	M80.0-M82.9	L95…	N33… ,

For peer review only - http://bmjopen.bmj.com/site/about/guidelines.xhtml

42	Other blood vessel disease (Raynaud's disease, Burger's disease)	I73.0-I73.9	K92…	G73… ,
43	Parkinson's disease	G20.9	N87-N87.01	F12…,
44	Peripheral vascular disease (Atherosclerosis)	170.0-170.9	K91	G70…, G71… , G72…
45	Polymyalgia	M35.3	L99.12	N20.11
46	Psoriasis	L40.0-L41.9	S91	M161…
47	Psoriatic arthritis	M07.0-M07.3	L99.13	M160…
48	Rheumatoid Arthritis	M05.0-M05.9	L88… , K71…	N04….
49	Renal stones	N20.0 ….	U95	4G4… , 7B07… , KB12…
50	Schizophrenia and/or psychosis	F20.0-F20.9, F25.0- F25.9	P72	E10…
51	Severe allergy			H17… , SN5…
52	Sjögren's syndrome	M35.0	NA	N002…
53	Systemic Lupus Erythematosus	M32.0, M32.1, M32.8, M32.9	NA	N000…
54	Sleep disorder (Insomnia)	F51.0	P06	Fy0… , 1B1B…
55	Solid malignancy	C00.0-C80.9, D00.0-D09.9, C97.9	A29… , A79, B74 – Y78	B0… - B67… , Byu…
56	Stroke	G45.0-G46.8, I60.0-I63.9, I65.0-166.9, I69.0-I69.4	K89-K90.02	G60… - G68…, F22…
57	Substance abuse/ Drug addiction	F10.0-F19.9	P18, P19…	E24…, Eu1…
58	Thrombotic diseases	I74.0-I74.9	K93, K94…, W99.03	G80… , G81… , G74…
59	Tuberculosis	A15.0-A16.9, B90.9	A70, R70	A1…, A11…
60	Vertigo	H81.4 …	N17-N17.02, H82-H82.03	R004… , F561…
61	Vision problem (Glaucoma and other)	H27.0-H27.9, H40.0-H40.9, H42.0-H42.8	F93…, F94	F45…, F49… ,

ICD- International Classification of Diseases; ICPC- International classification in primary care. All the codes are the primary code initials used in the database.

For peer review only

Table 4. Group of conditions/Outcome

Group	Conditions
Cardiovascular	Cardiac arrhythmias, Coronary Heart Disease (including AMI, valvular disease, angina), Heart failure, Hypertension, Peripheral vascular disease (claudication, Raynaud syndrome, Buerger's disease), Other blood vessel disease (atherosclerosis and aneurysm), Thrombotic diseases
Gastrointestinal	GERD (Esophageal diseases, gastritis, duodenitis), GI bleeding, Inflammatory bowel disease (IBD), Irritable bowel syndrome (IBS)
Musculoskeletal	Ankylosing spondylitis, Chronic Back pain, Chronic Neck pain, Fibromyalgia, Polymyalgia, Gout, Osteoporosis, Psoriatic arthritis, Rheumatoid arthritis, Sjögren's syndrome, Systemic lupus erythematosus (SLE)
Endocrine	Diabetes mellitus, Dyslipidemia (hyper), Hyperthyroidism, Hypothyroidism
Neurological	Dementia, Epilepsy, Fatigue, Migraine, Multiple sclerosis, Parkinson disease, Stroke
Psychological	Anxiety, Depression, Eating disorders (Anorexia / Bulimia nervosa), Schizophrenia, Sleep disorder (insomnia),
Kidney disease	Chronic kidney disease (any cause), Renal stones
Liver diseases	Gall bladder stone, Hepatitis, Liver cirrhosis
Respiratory	Asthma, Chronic obstructive pulmonary disease (COPD)
Cancer	Leukemia, Lymphoma, Solid Malignancy (any type)
Others	Anemia (all types), Benign prostate hypertrophy (BPH), Cataract, Chronic sinusitis, Eczema/Skin disease, Hearing impairment (all types), Psoriasis, Severe allergy (anaphylactic shock), angioneurotic oedema

For peer review only

Figure 1. Overview of the study design and statistical analysis plan for the Comorbidities in Osteoarthritis (CoMO) study.

	Objective 1	Objective 2	Objective 3	Objective 4
Study Design	Case-control and Cohort	Clustering and longitudinal	Cohort	Genomic association
Exposure	OA	OA	Analgesics (NSAIDS, Opioids, paracetamol)	OA
Outcome	Comorbidities	Clusters of comorbidities	Comorbidities	Comorbidities
Statistical methods	Conditional logistic regression, Cox regression	Latent class analysis, Latent class growth analysis Joint latent class analysis	Cox regression Time varying analysis Flexible parametric method	Mendelian randomisation
Reported Outcome	Odds Ratio and Hazard Ratio	Clusters and groups	Hazard Ratio	Coefficients
Participating centres	ALL	ALL	ALL	UK and Netherlands

Objective 5 — Meta Analysis

OA- osteoarthritis; NSAIDs- Nonsteroidal anti-inflammatory drugs

For peer review only

STROBE 2007 (v4) checklist of items to be included in reports of observational studies in epidemiology*
Checklist for cohort, case-control, and cross-sectional studies (combined)

**Section/Topic**	**Item #**	**Recommendation**	**Reported on page #**
**Title and abstract**	1	**(*a*) Indicate the study's design with a commonly used term in the title or the abstract**<br>Comorbidities in Osteoarthritis (CoMO): a multinational study in four European countries	1-2
		**(*b*) Provide in the abstract an informative and balanced summary of what was done and what was found**<br>*Introduction*<br>Osteoarthritis (OA) is one of the leading chronic conditions in the older population. People with OA are more likely to have one or more other chronic conditions than those without. However, the temporal associations, clusters of the comorbidities, role of analgesics and the causality and variation between populations are yet to be investigated. This paper describes the protocol of a multinational study in four European countries (UK, Netherlands, Sweden, and Spain) exploring comorbidities in people with OA .<br>*Methods and analysis*<br>This multinational study will investigate i) the temporal associations of 61 identified comorbidities with OA, ii) the clusters and trajectories of comorbidities in people with OA, iii) the role of analgesics on incidence of comorbidities in people with OA, iv) the potential biomarkers and causality between OA and the comorbidities, and v) variations between countries.<br>A combined case-control and cohort study will be conducted to find the temporal association of OA with the comorbidities using the national or regional health databases. Latent class analysis will be performed to identify the clusters at baseline and joint latent class analysis will be used to examine trajectories during the follow-up. A cohort study will be undertaken to evaluate the role of non-steroidal anti-inflammatory drugs (NSAIDs), opioids, and paracetamol on the incidence of comorbidities. Mendelian randomisation will be performed to investigate the potential biomarkers for causality between OA and the comorbidities using the UK Biobank and the Rotterdam	1

For peer review only

		Study databases. Finally, a meta-analyses will be used to examine the variations and pool the results from different countries.<br>*Ethics and Dissemination*<br>Research ethics was obtained according to each database requirement. Results will be disseminated through the FOREUM website, scientific meetings, publications, and in partnership with patient organisations.	
**Introduction**			
Background/rationale	2	**Explain the scientific background and rationale for the investigation being reported**<br>Osteoarthritis (OA) affects 27% of people aged over 45 years at peripheral synovial joints such as knees, hips, hands and feet (1). It is by far the most common form of arthritis, and a leading cause of chronic joint pain and disability in older people (2,3). It is anticipated that the burden of OA will continue to rise in the coming decades because of population ageing and the increasing obesity prevalence – two major risk factors for OA (4,5). Co-occurrence of multiple chronic conditions in an individual with ageing is becoming a norm and OA is not an exception to this.<br>A recent systematic review has confirmed that people with OA are more likely to have other diseases, especially stroke, peptic ulcer, hypertension, and depression (6). Vast majority of these studies focused on additional presence (comorbidity) of cardiovascular and musculoskeletal conditions only (7–9). Whether these comorbidities just co-exist with OA, share common risk factors with OA, or are causes or consequences of OA remains largely unknown. There was also reporting of wide heterogeneity in definitions of OA and other chronic conditions, diagnosis and recording of diseases, sample sizes, and number of diseases studied in previously published studies included in the review (6). This diversity made the comparison and pooled estimation of comorbidity prevalence difficult.<br>Comorbidity in OA can occur due to multiple factors. Various hypotheses have been used to explain the existence of comorbidities in general, the most accepted of which are the concordant (diseases sharing similar	4-5

For peer review only

		pathophysiological risk factors) and discordant (diseases not sharing similar pathophysiological risk factors) theories (10). Additionally, prescription of drugs is also reported to be associated with comorbidity and multimorbidity (11). Especially in people with OA, the prescription of analgesics is common, and is associated with increased risk of other conditions such as cardiovascular, gastrointestinal and chronic renal diseases (12,13). Also, having multiple chronic conditions increases the chances of polypharmacy which further escalates the risk of other conditions.<br>OA is one of the leading conditions reported in multimorbidity research. Exploring the association of OA with other diseases would help in further explaining the burden and pattern of the comorbidity (14). However, the major issue in OA comorbidity research is the low number and specific types of conditions studied (16). Therefore, it is important to develop a consensus on both the count and typology of conditions to be studied to enable comparisons across populations and to derive pooled estimates as appropriate. Further, using uniform methods and definitions of diseases in computing these estimates would reduce heterogeneity and make the comparison more reliable. Understanding the temporal association with comorbidity and disease trajectory is crucial for any chronic condition, and this is possible through studies using longitudinal databases (15). However, one of the limitations of using observational data is that causal associations are difficult to establish, due to the interference of known and unknown confounders. In this study we have used the more recently developed method 'Mendelian Randomization', that can determine causal estimates through combining the use of genetic data and instrumental variable methods."<br>The burden of diseases in primary care often depends on the population structure, health infrastructure and individual factors such as income and education. Such factors vary between countries, because of the heterogeneity mentioned above there are no robust data comparing OA and its comorbidities between countries. Therefore, the aims of this study were to explore the burden, pattern, and causal factors of comorbidities in people with OA across four European countries using national registration databases in the UK, the Netherlands, Sweden, and	

For peer review only

		Spain.	
Objectives	3	**State specific objectives, including any pre-specified hypotheses**<br>1. To estimate the prevalence, incidence, and time sequence of comorbidities in OA<br>2. To examine the clusters of comorbidities and trajectories of clusters in OA and associations with death<br>3. To investigate the associations between commonly used OA drugs, such as non-steroidal anti-inflammatory (NSAIDs) and opioids, and risk of comorbidities<br>4. To identify the potential biomarkers and causal pathways between OA and the comorbidities<br>5. To examine the variations of OA comorbidities and clusters across countries.	5
**Methods**			
Study design	4	**Present key elements of study design early in the paper**<br>Figure 1	Figure 1
Setting	5	**Describe the setting, locations, and relevant dates, including periods of recruitment, exposure, follow-up, and data collection**<br>*Databases*<br>Four routinely collected national (the UK and the Netherlands) or regional (Sweden and Spain) health databases will be used for objectives 1-3. In addition, for objective 4, genomic associations of OA with comorbidities will be examined using two cohort studies from the UK (UK Biobank) (17) and the Netherlands (Rotterdam study) (18). The four national representative and regional databases contain information about the population with primary care consultations in four different countries. The longitudinal databases provide information about the diagnosis of the diseases by the general practitioners and some diagnoses made in secondary care, prescription of drugs, deaths, and other health utilisation indicators. The details of the databases and their properties are given in Table 1 and 2.<br>*Participants*<br>People registered with the respective databases aged 18 year or above are eligible for the study.	5-6

For peer review only

Participants	6	(*a*) *Cohort study*—Give the eligibility criteria, and the sources and methods of selection of participants. Describe methods of follow-up<br>*Case-control study*—Give the eligibility criteria, and the sources and methods of case ascertainment and control selection. Give the rationale for the choice of cases and controls	n/a
		**(*b*) *Cohort study*—For matched studies, give matching criteria and number of exposed and unexposed**<br>***Case-control study*—For matched studies, give matching criteria and the number of controls per case**<br><br>*Objective 1 – Prevalence, incidence, and time sequence of comorbidities in OA*<br><br>A combined retrospective and prospective study of OA cases and sex, age (+/-2 years), first year of registration, and practice matched controls (1:1-4) without OA (28) will be used to determine the prevalence, incidence, and time sequence of comorbidities in OA. Incident OA cases will be identified, and the first diagnosis date will be used as the starting point (index date). For controls the same index date as their matched case will be used. They will be both retrospectively reviewed for prior diagnoses of comorbidities and prospectively followed-up for posterior new comorbidities.<br>For the prospective analysis participants with incident OA but without the specific comorbidity of interest at the index date (i.e., people at risk) and matched controls without OA will be followed up until the date of the first diagnosis of the comorbidity, deregistration, or death whichever comes first. The cumulative incidence will be calculated for each comorbidity in OA cases and matched controls at 1, 3, 5, 10, 15, 20 years (based on the data available) after the index date to examine the dynamic change of developing comorbidities during follow-up.<br>*Objective 3*<br>A cohort study will be undertaken for this objective to evaluate the contribution of common analgesics for OA to the development of comorbidity such as NSAIDs, opioids and paracetamol. We are interested in the interaction between OA and use of drugs on the incidence of comorbidities, i.e. to evaluate if the drug use in persons with OA poses increased or decreased risk of comorbidities compared to persons without OA and/or analgesics. Individual	7-8<br><br>9

For peer review only

		comorbidity, as well as clusters of comorbidities identified from Objective 2 will be examined as outcomes. The 61 comorbidities in our study will be further categorised into eight groups, specifically: musculoskeletal (MSK), respiratory, neurodegenerative, psychological/psychiatric, cancer, cardiovascular, metabolic, renal problem, liver diseases, gastrointestinal (GI) and others. (Table 4) The prospective cohort established from Objective 1 will form the source population for this objective. Individuals with incident OA will be identified from the database and the first diagnosis date will be used as index date for follow-up. Individuals without OA during the study period will be selected and matched with cases by age, sex, and practice. The same index date will be given from their matched OA cases. Individuals with analgesics prescriptions prior to the index date will be excluded (or recorded as a confounding factor to be adjusted as appropriate). Only analgesic prescriptions after the index date will be considered for this analysis. Prescriptions will be quantified as number of prescriptions within year 1 (initial use, primary analysis) (33), 2, 3, 4, 5 etc. It will also be dichotomised as episodic (e.g., at least one gap of ≥90 days between prescriptions) and continuous (no gap of more less than 90 days) users as appropriate (34,35). Analgesic use will be included in the model as a risk factor together with OA diagnosis (yes/no, primary exposure) to examine the independent risk of each variable (OA and analgesics), as well as the interaction between the two to the development of comorbidity. Dose response relationship will be examined using number of prescriptions during the exposure window examined. The effect of stopping analgesics will also be examined by looking into the patterns of analgesic prescriptions, e.g., stopping analgesics after initial use in year 1 versus continuous use of analgesics afterwards.<br>*Objective 4*<br>We will use the Rotterdam Study and the UK Biobank jointly for this objective. This is because that the Rotterdam Study is an OA cohort with deep phenotypes and biomarkers of OA, whereas the UK Biobank is a primary cohort for cancer and multiple disease outcomes, and both have detailed genetic variants. We will use two sample MR	10

For peer review only

		approach, i.e., to establish an association between OA and genetic variants in the Rotterdam Cohort to identify genetic instrumental variables (IV), e.g., a set of single nucleotide polymorphisms (SNPs) associated with OA (or a deep phenotype, biomarker, or risk factor of OA). We will then undertake the MR-PheWAS analysis to examine the causal effects of the OA IV on comorbidities in the UK Biobank. The MR method has been widely used in real world data to examine the causal relationship between IV and specific disease, under two assumptions: [1] genetic variants are randomly assigned in the population; and [2] genetic variants can only be the cause not consequence of disease (39). The PheWAS is a series of case control studies to estimate the associations between the IV and multiple disease outcomes (38,40). The combination of the two permits investigation of the causal effects of OA on multiple disease outcomes.	
Variables	7	**Clearly define all outcomes, exposures, predictors, potential confounders, and effect modifiers. Give diagnostic criteria, if applicable**<br>*Definition of Osteoarthritis*<br>OA will be defined as having at least one recorded physician diagnosis of OA for hip, knee, ankle/foot, wrist/hand, or site recorded as 'unspecified' during the study period for the respective database. People with any previous recording of the OA prior to the start date of the study will be excluded.<br>*Comorbidities*<br>We defined comorbidity as the recording of diagnosis of predefined chronic conditions in individuals using either ICD-10 or Read or international classification of primary care (ICPC) code. An extensive list of 61 chronic conditions was prepared from the Quality Outcome Framework (QOF) (19), list of the US Department of Health and Human Services Initiative on Multiple Chronic Conditions (20), global burden of diseases (21) and the Charlson comorbidity index (22). The list has been updated with findings from our systematic review (6) and a previous UK community-based knee pain study (6,23) by including common and important morbidities not included in the above (24,25). A code mapping exercise was conducted to finalise the list of conditions available for all the research centres. The comparison of codes was made, and it was reviewed by four researchers including	6-7

For peer review only

		a clinician from the team. The detailed list of the conditions is given in Table 3.<br>*Covariates*<br>Age and sex will be used in all centres as covariates to adjust in regression models. Additionally, information on body mass index (BMI), smoking, alcohol use, socioeconomic variables such as education level, income, place of birth (to identify those who immigrated to the country), and residential area, marriage (or registered partner) will be included when available. For calculating severity of the comorbidities in an individual, Elixhauser comorbidity index will be used to estimate the impact of comorbidities on death (26,27). Missing data on covariates will be substituted using multiple imputation methods, provided that the data is missing at random, if applicable.	
Data sources/ measurement	8*	**For each variable of interest, give sources of data and details of methods of assessment (measurement). Describe comparability of assessment methods if there is more than one group**<br>*Databases*<br>Four routinely collected national (the UK and the Netherlands) or regional (Sweden and Spain) health databases will be used for objectives 1-3. In addition, for objective 4, genomic associations of OA with comorbidities will be examined using two cohort studies from the UK (UK Biobank) (17) and the Netherlands (Rotterdam study) (18). The four national representative and regional databases contain information about the population with primary care consultations in four different countries. The longitudinal databases provide information about the diagnosis of the diseases by the general practitioners and some diagnoses made in secondary care, prescription of drugs, deaths, and other health utilisation indicators. The details of the databases and their properties are given in Table 1 and 2.<br><br>*Data Harmonisation:*<br>Firstly, we carried out a code mapping exercise for identification of people with osteoarthritis (OA) and other comorbidities. We developed a list of chronic conditions and each centres shared the list of codes to be used for the conditions, such as Read code in CPRD (UK), ICPC2 (Rotterdam) and ICD-10 for Lund and Spanish as per their database. The code lists were compared and edited to maintain the uniformity. The list was screened by verified by	*5*<br><br><br><br><br><br><br><br><br><br>*7*

For peer review only

		two researchers and two GPs. We also used unform definition for inclusion of condition e.g. at least one recording of the chronic conditions. Because all the centres did not have all the listed comorbidities, a minimum number of chronic conditions and covariates common in all the database were identified to be studied. Similarly, we decided to have a minimum follow-up study of 5 years and centres with more registration period can use the entire length of data available.	
Bias	9	Describe any efforts to address potential sources of bias	Explained under each objective
Study size	10	Explain how the study size was arrived at<br>*Feasibility and sample size*<br>To detect minimum incidence of 1% comorbidity (required for cluster analysis) with a minimum clinical important difference of hazard ratio (HR)1.2, and 90% power of the study, the estimated sample size was 197561 for 1581 events. It was calculated using STATA, with a correlation= 0.2, standard deviation of 0.5, proportion of withdrawal= 0.20, alpha=0.05. The initial check with the registry database revealed to have minimum required sample size for the study.	11-12
Quantitative variables	11	Explain how quantitative variables were handled in the analyses. If applicable, describe which groupings were chosen and why	Explained under each objective
Statistical methods	12	**(*a*) Describe all statistical methods, including those used to control for confounding**<br>*Objective 1*<br>In the retrospective analysis the prevalence and 95% confidence interval (CI) of each specific comorbidity will be calculated separately in OA cases and matched controls using the number of people diagnosed with the comorbidity divided by the total number of OA cases or controls at the index date. The prevalence of each	8

For peer review only

		comorbidity in OA cases and matched controls will be calculated for given time intervals prior to the index date of 0-1, 0-5 and 0-10 years separately to assess observational bias (28). Discrete time intervals of 1-5, and 5-10 years before will also be used to estimate the prevalence to minimise consultation bias/misclassification bias of OA (if possible). Logistic regression will be used to calculate the odds ratios (OR) for each comorbidity unadjusted and adjusted for BMI, smoking and alcohol consumption.<br>Kaplan-Meier survival curves will be used to display the cumulative probability in OA and non-OA groups. Proportional hazard assumption will be tested using Schoenfeld residual plots. The Cox regression model will be used to calculate hazard ratios (HR) for each comorbidity unadjusted and adjusted for age, sex, practice, BMI, smoking and alcohol consumption. This hybrid design has been previously used by us to examine the temporality of associations between other rheumatic musculoskeletal diseases (RMDs) (e.g., gout and lupus) and comorbidities (28,29).<br><br>*Objective 2*<br><br>For each dataset, an 80%: 20% split into the training and testing data will be introduced. The following analysis in objective 2 will be first employed into the training dataset and then tested its generalisability in the testing dataset. At baseline, clusters of people based on 61 comorbidities will be identified using Latent class (i.e., Gaussian mixture models algorithms of cluster) analysis (30). For each model, we will examine the association between clusters and covariates using multinomial logistic regressions. The distinctness of clusters will be examined by comparing covariates among clusters. The optimal model is the one where most clusters found in the training data are also identified independently in the testing data and clusters have most distinct patients' characteristics. We will then use both latent trajectory analysis, such as joint latent class models (31), and unsupervised machine learning approach, such as deep autoencoder or recurrent neural networks (32), to identify distinct clusters of new	8<br><br>9

For peer review only - http://bmjopen.bmj.com/site/about/guidelines.xhtml

		comorbidity numbers development over time and their association with mortality with adjustment for baseline covariates.	
		Objective 3	
		For the primary analysis (initial prescriptions within year 1), a landmark analysis will be used to minimise the immortal time bias where the follow up will start after 12 months from the index date (36). Participants at risk (i.e., without a specific comorbidity of interest) at the landmark date will be followed up until the first diagnosis of the comorbidity, deregistration, or death whichever comes first. For secondary analyses, time varying covariate analysis will be used to examine the long-term, episodic/continuous use of analgesics after the index date and interaction between OA and analgesics in the development of the comorbidity. The propensity score matching or the inverse probability weighing methods will be used to adjust for confounding by indication during the follow-up as appropriate. Depending on the country-specific drug use patterns, we may modify this definition to allow for short brakes in between the episodes. Cox-regression model will be used to calculate the HR and 95% CI. We will use flexible parametric models using restricted cubic splines (developed by Lambert, "stpm2" in Stata) to estimate the HRs and differences in time to diagnosis of comorbidities (outcome) with drugs as time-varying to account for non-proportional hazards (37).	10
		Objective 4	
		The MR-PheWAS analysis includes three steps. Firstly, we will identify the genetic variants that are associated with OA - IV. Secondly, we will undertake the PheWAS analysis – a series of case control analyses to estimate the associations between the IV and other disease outcomes (38,40) , with an adjustment for multiple testing using the false discovery rate (FDR) methods (41). Thirdly, we will implement conventional MR analysis to investigate the causal effects of the OA IV on comorbidities (39). An inverse variance weighted (IVW) method will be used to pool the associations (ORs) as appropriate (42). The MR-Egger regression analysis will be used to count for the pleiotropic effect - the effects of one genetic variant on multiple outcomes (43). The heterogeneity in dependent	10-11

For peer review only

		instruments (HEIDI) test will be used to exclude the cross-phenotype associations caused by genetic linkage (44).<br><br>With the MR-PheWAS study, the OR can be interpreted as causal association. We are primarily interested in the causality from OA to comorbidities. We are also interested in inflammatory (e.g., CRP), metabolic (e.g., gut microbiome) and biomechanics (e.g., BMI) biomarkers and deep phenotypes of OA such as knee, hip, and hand OA with and without symptoms. This will be undertaken if it is feasible within 3 years of this funded project, otherwise will be considered as our future research agenda.<br><br>*Objective 5*<br><br>We will use meta-analyses (MA) to examine the variation between countries and to pool the data as appropriate. Estimates from first three objectives such as prevalence, incidence, OR, HR and 95% CI for each specific comorbidity across different populations will be distributed in a forest plot. Heterogeneity will be examined using the I^2 statistic and the Q test (45). Results will be pooled if they are homogenous based on the I^2 value using the fixed effects model, otherwise the reasons for the heterogeneity will be investigated. Random effects models will be used to pool the results if the reasons for the heterogeneity cannot be identified and if the overall pooling is appropriate. Individual patient data (IPD) meta-analysis may be used to help identify the reasons for heterogeneity (46). Common clusters and trajectories as well as burdens of comorbidities will also be compared between populations.	11
		**(*b*) Describe any methods used to examine subgroups and interactions**<br>Please see method sections	n/a
		**(*c*) Explain how missing data were addressed**<br>Missing data on covariates will be substituted using multiple imputation methods, provided that the data is missing at random, if applicable.	7
		(*d*) *Cohort study*—If applicable, explain how loss to follow-up was addressed<br>*Case-control study*—If applicable, explain how matching of cases and controls was addressed<br>*Cross-sectional study*—If applicable, describe analytical methods taking account of sampling strategy	n/a
		(*e*) Describe any sensitivity analyses	n/a

For peer review only

**Results**			
Participants	13*	(a) Report numbers of individuals at each stage of study—eg numbers potentially eligible, examined for eligibility, confirmed eligible, included in the study, completing follow-up, and analysed	n/a
		(b) Give reasons for non-participation at each stage	n/a
		(c) Consider use of a flow diagram	n/a
Descriptive data	14*	(a) Give characteristics of study participants (eg demographic, clinical, social) and information on exposures and potential confounders	n/a
		(b) Indicate number of participants with missing data for each variable of interest	n/a
		(c) *Cohort study*—Summarise follow-up time (eg, average and total amount)	n/a
Outcome data	15*	*Cohort study*—Report numbers of outcome events or summary measures over time	n/a
		Case-control study—Report numbers in each exposure category, or summary measures of exposure	n/a
		Cross-sectional study—Report numbers of outcome events or summary measures	n/a
Main results	16	(*a*) Give unadjusted estimates and, if applicable, confounder-adjusted estimates and their precision (eg, 95% confidence interval). Make clear which confounders were adjusted for and why they were included	n/a
		(*b*) Report category boundaries when continuous variables were categorized	n/a
		(*c*) If relevant, consider translating estimates of relative risk into absolute risk for a meaningful time period	n/a
Other analyses	17	Report other analyses done—eg analyses of subgroups and interactions, and sensitivity analyses	n/a
**Discussion**			
Key results	18	Summarise key results with reference to study objectives	n/a
Limitations	19	**Discuss limitations of the study, taking into account sources of potential bias or imprecision. Discuss both direction and magnitude of any potential bias**<br>There are some limitations to this study. Firstly, there are inherent issues in the nature of electronic health records with respect to possible misdiagnosis, ascertainment biases, under- or over-recording, and changes in databases due to change in coding structures. Also, the analysis will be restricted to fewer covariates in some databases due to missing information on lifestyle factors such as physical activities and diet. Even though the databases have different durations of data available, if possible we will use a common follow-up time for objective five. Another important limitation is that we do not have information on quality of life and other outcomes to measure functional limitations recorded in the database.<br>Chronic conditions, especially comorbidities recorded in general practices, depend on multiple factors such as population structure, health care facilities, health policies, and the nature of the national databases. A major	13

For peer review only

		strength of this study is that it will include medical records on approximately 27 million people in four European countries. Also, the study will cover the sequence of research questions in comorbidity or multimorbidity starting from the burden through to the causality and variation. Such a research model can be used for other similar multimorbidity studies. The expected results should inform health professionals in primary care settings with respect to management of people with OA and associated comorbidities.	
Interpretation	20	Give a cautious overall interpretation of results considering objectives, limitations, multiplicity of analyses, results from similar studies, and other relevant evidence	n/a
Generalisability	21	Discuss the generalisability (external validity) of the study results	n/a
**Other information**			
Funding	22	**Give the source of funding and the role of the funders for the present study and, if applicable, for the original study on which the present article is based** This work was supported by Foundation for Research in Rheumatology (FOREUM) grant (2019-2022), The Swedish Research Council (2020-01103), Governmental funding of clinical research within the national health services (ALF), and The Swedish Rheumatism Association. CM is funded by the National Institute for Health Research (NIHR) Applied Research Collaboration West Midlands, the National Institute for Health Research (NIHR) School for Primary Care Research and a National Institute for Health Research (NIHR) Research Professorship in General Practice (NIHR-RP-2014-04-026)	14

*Give information separately for cases and controls in case-control studies and, if applicable, for exposed and unexposed groups in cohort and cross-sectional studies.
Note: An Explanation and Elaboration article discusses each checklist item and gives methodological background and published examples of transparent reporting. The STROBE checklist is best used in conjunction with this article (freely available on the Web sites of PLoS Medicine at http://www.plosmedicine.org/, Annals of Internal Medicine at http://www.annals.org/, and Epidemiology at http://www.epidem.com/). Information on the STROBE Initiative is available at www.strobe-statement.org.

For peer review only

BMJ Open

Comorbidities in Osteoarthritis (ComOA): a combined cross-sectional, case-control and cohort study using large electronic health records in four European countries

Journal:	*BMJ Open*
Manuscript ID	bmjopen-2021-052816.R2
Article Type:	Protocol
Date Submitted by the Author:	15-Feb-2022
Complete List of Authors:	Swain, Subhashisa; University of Nottingham, Academic Rheumatology; University of Oxford, Nuffield Department of Primary Care Health Sciences<br>Kamps, Anne; Erasmus Medical Center Department of Reumatology, Department of General Practice<br>Runhaar, Jos; Erasmus Medical Center Department of Reumatology, Department of General Practice<br>Dell'Isola, Andrea; Lunds University Faculty of Medicine,<br>Turkiewicz, Aleksandra; Lund University Faculty of Medicine, Dept of Orthopedics<br>Robinson, Danielle ; University of Oxford Nuffield Department of Orthopaedics Rheumatology and Musculoskeletal Sciences,<br>Strauss, V; University of Oxford Nuffield Department of Orthopaedics Rheumatology and Musculoskeletal Sciences,<br>Mallen, Christian; Keele University, Arthritis Research UK Primary Care Centre<br>Kuo, Chang-Fu; Chang Gung Memorial Hospital Linkou Branch,<br>Coupland, Carol; University of Nottingham, Division of Primary Care<br>Doherty, Michael ; University of Nottingham School of Medicine, Academic Rheumatology<br>Sarmanova, Aliya ; University of Bristol,<br>Prieto-Alhambra, Daniel; University of Oxford, Centre for Statistics in Medicine, NDORMS<br>Englund, Martin; Lund University, Dept of Orthopedics<br>Bierma-Zeinstra, Sita; Erasmus University Medical Centre, Department of General Practice<br>Zhang, Weiya; University of Nottingham School of Medicine, Academic Rheumatology
**Primary Subject Heading**:	Rheumatology
Secondary Subject Heading:	Epidemiology, Health services research, Public health, Pharmacology and therapeutics, Rheumatology
Keywords:	EPIDEMIOLOGY, Musculoskeletal disorders < ORTHOPAEDIC & TRAUMA SURGERY, RHEUMATOLOGY

SCHOLARONE™
Manuscripts

Title- Comorbidities in Osteoarthritis (ComOA): a combined cross-sectional, case-control and cohort study using large electronic health records in four European countries

Authors

Subhashisa Swain[1,2], Anne Kamps[3], Jos Runhaar[3], Andrea Dell 'Isola[4], Aleksandra Turkiewicz[4], Danielle Robinson[5], Victoria Y Strauss[5], Christian Mallen[6], Chang Fu Kuo[1,7], Carol Coupland[8], Michael Doherty[1,11], Aliya Sarmanova[9], Daniel Prieto Alhambra[5], Martin Englund[4], Sita Bierma-Zeinstra[10], Weiya Zhang[1,11]

1. Academic Rheumatology, School of Medicine, University of Nottingham, UK
2. Nuffield Department of Primary Care Health Sciences, University of Oxford, UK
3. Department of General Practice, Erasmus MC University Medical Center Rotterdam, The Netherlands
4. Clinical Epidemiology Unit, Orthopaedics, Department of Clinical Sciences Lund, Lund University, Sweden
5. Centre for Statistics in Medicine, NDORMS, University of Oxford, UK
6. School of Medicine, Keele University, UK
7. Division of Rheumatology, Allergy and Immunology, Chang Gung Memorial Hospital, Taiwan.
8. Division of Primary Care, School of Medicine, University of Nottingham, UK
9. Musculoskeletal Research Unit, Bristol Medical School, Translational Health Sciences, University of Bristol, UK
10. Department of General Practice, Department of Orthopaedic Surgery & Sports Medicine, Erasmus MC University Medical Center Rotterdam, The Netherlands
11. Pain Centre Versus Arthritis, University of Nottingham, UK

Corresponding Author:

Professor Weiya Zhang

Academic Rheumatology, School of Medicine

University of Nottingham

Clinical Sciences Building, Nottingham city Hospital

NG5 1PB, Nottingham, UK

Email: weiya.zhang@nottingham.ac.uk

For peer review only

For peer review only - http://bmjopen.bmj.com/site/about/guidelines.xhtml

Abstract

Introduction

Osteoarthritis (OA) is one of the leading chronic conditions in the older population. People with OA are more likely to have one or more other chronic conditions than those without. However, the temporal associations, clusters of the comorbidities, role of analgesics and the causality and variation between populations are yet to be investigated. This paper describes the protocol of a multinational study in four European countries (UK, Netherlands, Sweden, and Spain) exploring comorbidities in people with OA .

Methods and analysis

This multinational study will investigate i) the temporal associations of 61 identified comorbidities with OA, ii) the clusters and trajectories of comorbidities in people with OA, iii) the role of analgesics on incidence of comorbidities in people with OA, iv) the potential biomarkers and causality between OA and the comorbidities, and v) variations between countries.

A combined case-control and cohort study will be conducted to find the temporal association of OA with the comorbidities using the national or regional health databases. Latent class analysis will be performed to identify the clusters at baseline and joint latent class analysis will be used to examine trajectories during the follow-up. A cohort study will be undertaken to evaluate the role of non-steroidal anti-inflammatory drugs (NSAIDs), opioids, and paracetamol on the incidence of comorbidities. Mendelian randomisation will be performed to investigate the potential biomarkers for causality between OA and the comorbidities using the UK Biobank and the Rotterdam Study databases. Finally, a meta-analyses will be used to examine the variations and pool the results from different countries.

Ethics and Dissemination

Research ethics was obtained according to each database requirement. Results will be disseminated through the FOREUM website, scientific meetings, publications, and in partnership with patient organisations.

Key words: Osteoarthritis; Comorbidity; Multimorbidity; Primary care; Analgesics, Mendelian Randomisation, PheWAS

For peer review only - http://bmjopen.bmj.com/site/about/guidelines.xhtml

Strengths and limitations of this study

- This is first ever multicenter study on comorbidities in osteoarthritis in Europe involving nearly 27 million electronic health records
- More than 60 chronic conditions are being studied – representing a wider coverage of diseases
- We will examine the causal association using Mendelian Randomization -PheWAS methods with genetic data collected from two countries
- Same protocol with robust statistical methods will be used across all countries to replicate the findings and examine the variations.
- Possible biases may be introduced by the nature of electronic health records and length of data availability.

For peer review only

Background

Osteoarthritis (OA) affects 27% of people aged over 45 years at peripheral synovial joints such as knees, hips, hands and feet (1). It is by far the most common form of arthritis, and a leading cause of chronic joint pain and disability in older people (2,3). It is anticipated that the burden of OA will continue to rise in the coming decades because of population ageing and the increasing obesity prevalence – two major risk factors for OA (4,5). Co-occurrence of multiple chronic conditions in an individual with ageing is becoming a norm and OA is not an exception to this.

A recent systematic review has confirmed that people with OA are more likely to have other diseases, especially stroke, peptic ulcer, hypertension, and depression (6). Vast majority of these studies focused on additional presence (comorbidity) of cardiovascular and musculoskeletal conditions only (7–9). Whether these comorbidities just co-exist with OA, share common risk factors with OA, or are causes or consequences of OA remains largely unknown. There was also reporting of wide heterogeneity in definitions of OA and other chronic conditions, diagnosis and recording of diseases, sample sizes, and number of diseases studied in previously published studies included in the review (6). This diversity made the comparison and pooled estimation of comorbidity prevalence difficult.

Comorbidity in OA can occur due to multiple factors. Various hypotheses have been used to explain the existence of comorbidities in general, the most accepted of which are the concordant (diseases sharing similar pathophysiological risk factors) and discordant (diseases not sharing similar pathophysiological risk factors) theories (10). Additionally, prescription of drugs is also reported to be associated with comorbidity and multimorbidity (11). Especially in people with OA, the prescription of analgesics is common, and is associated with increased risk of other conditions such as cardiovascular, gastrointestinal and chronic renal diseases (12,13). Also, having multiple chronic conditions increases the chances of polypharmacy which further escalates the risk of other conditions.

OA is one of the leading conditions reported in multimorbidity research. Exploring the association of OA with other diseases would help in further explaining the burden and pattern of the comorbidity (14). However, the major issue in OA comorbidity research is the low number and specific types of conditions studied (15). Therefore, it is important to develop a consensus on both the count and typology of conditions to be studied to enable comparisons across populations and to derive pooled estimates as appropriate. Further, using uniform

For peer review only - http://bmjopen.bmj.com/site/about/guidelines.xhtml

methods and definitions of diseases in computing these estimates would reduce heterogeneity and make the comparison more reliable.

Understanding the temporal association with comorbidity and disease trajectory is crucial for any chronic condition, and this is possible through studies using longitudinal databases (16). However, one of the limitations of using observational data is that causal associations are difficult to establish, due to the interference of known and unknown confounders. In this study we have used the more recently developed method 'Mendelian Randomization', that can determine causal estimates through combining the use of genetic data and instrumental variable methods."

The burden of diseases in primary care often depends on the population structure, health infrastructure and individual factors such as income and education. Such factors vary between countries, because of the heterogeneity mentioned above there are no robust data comparing OA and its comorbidities between countries. Therefore, the aims of this study were to explore the burden, pattern, and causal factors of comorbidities in people with OA across four European countries using national registration databases in the UK, the Netherlands, Sweden, and Spain.

Objectives

1. To estimate the prevalence, incidence, and time sequence of comorbidities in OA
2. To examine the clusters of comorbidities and trajectories of clusters in OA and associations with death
3. To investigate the associations between commonly used OA drugs, such as non-steroidal anti-inflammatory (NSAIDs) and opioids, and risk of comorbidities
4. To identify the potential biomarkers and causal pathways between OA and the comorbidities
5. To examine the variations of OA comorbidities and clusters across countries.

Methods

Databases

Four routinely collected national (the UK and the Netherlands) or regional (Sweden and Spain) health databases will be used for objectives 1-3. In addition, for objective 4, genomic associations of OA with comorbidities will be examined using two cohort studies from the UK (UK Biobank) (17) and the Netherlands (Rotterdam study) (18). The four national representative and regional databases contain information about the population with primary

For peer review only - http://bmjopen.bmj.com/site/about/guidelines.xhtml

care consultations in four different countries. The longitudinal databases provide information about the diagnosis of the diseases by the general practitioners and some diagnoses made in secondary care, prescription of drugs, deaths, and other health utilisation indicators. The details of the databases and their properties are given in Table 1 and 2.

Participants

People registered with the respective databases aged 18 year or above are eligible for the study.

Patient public involvement (PPI): Three PPI representatives (with OA and all with multiple chronic conditions) were involved in this study through group meetings. Difficulties of living with multiple conditions, lack of research in causal relationship and identification of diseases to be studied and the role of drugs in comorbidity were discussed. They are constantly in touch through providing their inputs at each step of the study.

Definition of Osteoarthritis

OA will be defined as having at least one recorded physician diagnosis of OA for hip, knee, ankle/foot, wrist/hand, or site recorded as 'unspecified' during the study period for the respective database. People with any previous recording of the OA prior to the start date of the study will be excluded.

Comorbidities

We defined comorbidity as the recording of diagnosis of predefined chronic conditions in individuals using either ICD-10 or Read or international classification of primary care (ICPC) code. An extensive list of 61 chronic conditions was prepared from the Quality Outcome Framework (QOF) (19), list of the US Department of Health and Human Services Initiative on Multiple Chronic Conditions (20), global burden of diseases (21) and the Charlson comorbidity index (22). The list has been updated with findings from our systematic review (6) and a previous UK community-based knee pain study (6,23) by including common and important morbidities not included in the above (24,25). A code mapping exercise was conducted to finalise the list of conditions available for all the research centres. The comparison of codes was made, and it was reviewed by four researchers including a clinician from the team. The detailed list of the conditions is given in Table 3.

Covariates

Age and sex will be used in all centres as covariates to adjust in regression models. Additionally, information on body mass index (BMI), smoking, alcohol use, socioeconomic variables such as education level, income, place of birth (to identify those who immigrated to

For peer review only - http://bmjopen.bmj.com/site/about/guidelines.xhtml

the country), and residential area, marriage (or registered partner) will be included when available. For calculating severity of the comorbidities in an individual, Elixhauser comorbidity index will be used to estimate the impact of comorbidities on death (26,27). Missing data on covariates will be substituted using multiple imputation methods, provided that the data is missing at random, if applicable.

Data Harmonisation:

Firstly, we carried out a code mapping exercise for identification of people with osteoarthritis (OA) and other comorbidities. We developed a list of chronic conditions and each centres shared the list of codes to be used for the conditions, such as Read code in CPRD (UK), ICPC2 (Rotterdam) and ICD-10 for Lund and Spanish as per their database. The code lists were compared and edited to maintain the uniformity. The list was screened by verified by two researchers and two GPs. We also used unform definition for inclusion of condition e.g. at least one recording of the chronic conditions. Because all the centres did not have all the listed comorbidities, a minimum number of chronic conditions and covariates common in all the database were identified to be studied. Similarly, we decided to have a minimum follow-up study of 5 years and centres with more registration period can use the entire length of data available.

Study design and data analysis

Summary of the study design and analysis is provided in Figure 1. All the centres plan to use same statistical analysis plan to investigate each objective.

Ethics and Dissemination:

The study has obtained the following ethics approvals: UK- Independent Scientific Advisory Council (ISAC) 19/30R, The Netherlands- The Integrated Primary Care Information (IPCI) registration no. 11/2019, Spain - the Information System for the Development of Research in Primary Care (SIDIAP), 4R19/011 , Sweden –Ethical Review Authority, Skåne Healthcare Register, 'Dnr 2011-432, Dnr 2014-276, and Dnr 2018-233. The registry databases are made available anonymised for the research purposes. Each centre will follow the data privacy policy of respective countries.

We plan to publish all the results as manuscripts in peer reviewed journals and present the findings in relevant conferences. The results would be circulated as 'lay-person' language and would be available at least four different international languages such as English, Dutch,

For peer review only - http://bmjopen.bmj.com/site/about/guidelines.xhtml

Swedish and Spanish. These will be shared on appropriate patient-public forum and involved institution's websites.

Objective 1 – Prevalence, incidence, and time sequence of comorbidities in OA

A combined retrospective and prospective study of OA cases and sex, age (+/-2 years), first year of registration, and practice matched controls (1:1-4) without OA (28) will be used to determine the prevalence, incidence, and time sequence of comorbidities in OA. Incident OA cases will be identified, and the first diagnosis date will be used as the starting point (index date). For controls the same index date as their matched case will be used. They will be both retrospectively reviewed for prior diagnoses of comorbidities and prospectively followed-up for posterior new comorbidities. In the retrospective analysis the prevalence and 95% confidence interval (CI) of each specific comorbidity will be calculated separately in OA cases and matched controls using the number of people diagnosed with the comorbidity divided by the total number of OA cases or controls at the index date. The prevalence of each comorbidity in OA cases and matched controls will be calculated for given time intervals prior to the index date of 0-1, 0-5 and 0-10 years separately to assess observational bias (28). Discrete time intervals of 1-5, and 5-10 years before will also be used to estimate the prevalence to minimise consultation bias/misclassification bias of OA (if possible). Logistic regression will be used to calculate the odds ratios (OR) for each comorbidity unadjusted and adjusted for BMI, smoking and alcohol consumption.

For the prospective analysis participants with incident OA but without the specific comorbidity of interest at the index date (i.e., people at risk) and matched controls without OA will be followed up until the date of the first diagnosis of the comorbidity, deregistration, or death whichever comes first. The cumulative incidence will be calculated for each comorbidity in OA cases and matched controls at 1, 3, 5, 10, 15, 20 years (based on the data available) after the index date to examine the dynamic change of developing comorbidities during follow-up. Kaplan-Meier survival curves will be used to display the cumulative probability in OA and non-OA groups. Proportional hazard assumption will be tested using Schoenfeld residual plots. The Cox regression model will be used to calculate hazard ratios (HR) for each comorbidity unadjusted and adjusted for age, sex, practice, BMI, smoking and alcohol consumption. This hybrid design has been previously used by us to examine the temporality of associations between other rheumatic musculoskeletal diseases (RMDs) (e.g., gout and lupus) and comorbidities (28,29).

For peer review only - http://bmjopen.bmj.com/site/about/guidelines.xhtml

Objective 2 – Clusters and impact of comorbidities in people with OA

For each dataset, an 80%: 20% split into the training and testing data will be introduced. The following analysis in objective 2 will be first employed into the training dataset and then tested its generalisability in the testing dataset. At baseline, clusters of people based on 61 comorbidities will be identified using Latent class (i.e., Gaussian mixture models algorithms of cluster) analysis (30). For each model, we will examine the association between clusters and covariates using multinomial logistic regressions. The distinctness of clusters will be examined by comparing covariates among clusters. The optimal model is the one where most clusters found in the training data are also identified independently in the testing data and clusters have most distinct patients' characteristics. We will then use both latent trajectory analysis, such as joint latent class models (31), and unsupervised machine learning approach, such as deep autoencoder or recurrent neural networks (32), to identify distinct clusters of new comorbidity numbers development over time and their association with mortality with adjustment for baseline covariates.

Objective 3 - Association between analgesics and incident comorbidities

A cohort study will be undertaken for this objective to evaluate the contribution of common analgesics for OA to the development of comorbidity such as NSAIDs, opioids and paracetamol. We are interested in the interaction between OA and use of drugs on the incidence of comorbidities, i.e. to evaluate if the drug use in persons with OA poses increased or decreased risk of comorbidities compared to persons without OA and/or analgesics. Individual comorbidity, as well as clusters of comorbidities identified from Objective 2 will be examined as outcomes. The 61 comorbidities in our study will be further categorised into eight groups, specifically: musculoskeletal (MSK), respiratory, neurodegenerative, psychological/psychiatric, cancer, cardiovascular, metabolic, renal problem, liver diseases, gastrointestinal (GI) and others. (Table 4) The prospective cohort established from Objective 1 will form the source population for this objective. Individuals with incident OA will be identified from the database and the first diagnosis date will be used as index date for follow-up. Individuals without OA during the study period will be selected and matched with cases by age, sex, and practice. The same index date will be given from their matched OA cases. Individuals with analgesics prescriptions prior to the index date will be excluded (or recorded as a confounding factor to be adjusted as appropriate). Only analgesic prescriptions after the index date will be considered for this analysis. Prescriptions will be quantified as number of prescriptions within year 1 (initial use, primary analysis) (33), 2, 3, 4, 5 etc. It will also be

For peer review only - http://bmjopen.bmj.com/site/about/guidelines.xhtml

dichotomised as episodic (e.g., at least one gap of ≥90 days between prescriptions) and continuous (no gap of more less than 90 days) users as appropriate (34,35). Analgesic use will be included in the model as a risk factor together with OA diagnosis (yes/no, primary exposure) to examine the independent risk of each variable (OA and analgesics), as well as the interaction between the two to the development of comorbidity. Dose response relationship will be examined using number of prescriptions during the exposure window examined. The effect of stopping analgesics will also be examined by looking into the patterns of analgesic prescriptions, e.g., stopping analgesics after initial use in year 1 versus continuous use of analgesics afterwards. For the primary analysis (initial prescriptions within year 1), a landmark analysis will be used to minimise the immortal time bias where the follow up will start after 12 months from the index date (36). Participants at risk (i.e., without a specific comorbidity of interest) at the landmark date will be followed up until the first diagnosis of the comorbidity, deregistration, or death whichever comes first. For secondary analyses, time varying covariate analysis will be used to examine the long-term, episodic/continuous use of analgesics after the index date and interaction between OA and analgesics in the development of the comorbidity. The propensity score matching or the inverse probability weighing methods will be used to adjust for confounding by indication during the follow-up as appropriate. Depending on the country-specific drug use patterns, we may modify this definition to allow for short brakes in between the episodes. Cox-regression model will be used to calculate the HR and 95% CI. We will use flexible parametric models using restricted cubic splines (developed by Lambert, "stpm2" in Stata) to estimate the HRs and differences in time to diagnosis of comorbidities (outcome) with drugs as time-varying to account for non-proportional hazards (37).

Objective 4 – Potential causal pathways between OA and the comorbidities

We will perform a Mendelian Randomisation (MR) phenome-wide association (MR-PheWAS) study(38) to examine the causal relationship between OA, its phenotypes, biomarkers or risk factors and comorbidities using the UK Biobank and the Rotterdam Study database.

We will use the Rotterdam Study and the UK Biobank jointly for this objective. This is because that the Rotterdam Study is an OA cohort with deep phenotypes and biomarkers of OA, whereas the UK Biobank is a primary cohort for cancer and multiple disease outcomes, and both have detailed genetic variants. We will use two sample MR approach, i.e., to establish an association between OA and genetic variants in the Rotterdam Cohort to identify

For peer review only - http://bmjopen.bmj.com/site/about/guidelines.xhtml

genetic instrumental variables (IV), e.g., a set of single nucleotide polymorphisms (SNPs) associated with OA (or a deep phenotype, biomarker, or risk factor of OA). We will then undertake the MR-PheWAS analysis to examine the causal effects of the OA IV on comorbidities in the UK Biobank. The MR method has been widely used in real world data to examine the causal relationship between IV and specific disease, under two assumptions: [1] genetic variants are randomly assigned in the population; and [2] genetic variants can only be the cause not consequence of disease (39). The PheWAS is a series of case control studies to estimate the associations between the IV and multiple disease outcomes (38,40). The combination of the two permits investigation of the causal effects of OA on multiple disease outcomes.

The MR-PheWAS analysis includes three steps. Firstly, we will identify the genetic variants that are associated with OA - IV. Secondly, we will undertake the PheWAS analysis – a series of case control analyses to estimate the associations between the IV and other disease outcomes (38,40) , with an adjustment for multiple testing using the false discovery rate (FDR) methods (41). Thirdly, we will implement conventional MR analysis to investigate the causal effects of the OA IV on comorbidities (39). An inverse variance weighted (IVW) method will be used to pool the associations (ORs) as appropriate (42). The MR-Egger regression analysis will be used to count for the pleiotropic effect - the effects of one genetic variant on multiple outcomes (43). The heterogeneity in dependent instruments (HEIDI) test will be used to exclude the cross-phenotype associations caused by genetic linkage (44).

With the MR-PheWAS study, the OR can be interpreted as causal association. We are primarily interested in the causality from OA to comorbidities. We are also interested in inflammatory (e.g., CRP), metabolic (e.g., gut microbiome) and biomechanics (e.g., BMI) biomarkers and deep phenotypes of OA such as knee, hip, and hand OA with and without symptoms. This will be undertaken if it is feasible within 3 years of this funded project, otherwise will be considered as our future research agenda.

Objective 5 – Variation of OA comorbidity patterns across countries

We will use meta-analyses (MA) to examine the variation between countries and to pool the data as appropriate. Estimates from first three objectives such as prevalence, incidence, OR, HR and 95% CI for each specific comorbidity across different populations will be distributed in a forest plot. Heterogeneity will be examined using the I^2 statistic and the Q test (45). Results will be pooled if they are homogenous based on the I^2 value using the fixed effects

For peer review only - http://bmjopen.bmj.com/site/about/guidelines.xhtml

model, otherwise the reasons for the heterogeneity will be investigated. Random effects models will be used to pool the results if the reasons for the heterogeneity cannot be identified and if the overall pooling is appropriate. Individual patient data (IPD) meta-analysis may be used to help identify the reasons for heterogeneity (46). Common clusters and trajectories as well as burdens of comorbidities will also be compared between populations.

Feasibility and sample size

To detect minimum incidence of 1% comorbidity (required for cluster analysis) with a minimum clinical important difference of hazard ratio (HR)1.2, and 90% power of the study, the estimated sample size was 197561 for 1581 events. It was calculated using STATA, with a correlation= 0.2, standard deviation of 0.5, proportion of withdrawal= 0.20, alpha=0.05. The initial check with the registry database revealed to have minimum required sample size for the study.

Discussion

This study will be the largest epidemiological study on comorbidities of OA in primary care. One of the key advantages of this multinational study is the use of the same protocol to measure the burden of comorbidities in primary care settings in four European countries to ensure reproducibility and comparison. There is scant evidence on the comorbidities in people with OA, and this approach should help to identify the leading and most important associations before and after presenting clinical OA (the index date). Further advantages of this study are the large and representative populations studied and the same/similar extensive list of chronic conditions for identifying comorbidity clusters. Often comorbidities accumulate with age over time and the large primary care databases in this study have the advantage of having long follow-up time which will enable us to detect the incidence of comorbidities. Also, longer follow up would help to identify the picture of the trajectory of the diseases (47). Both the incidence and the trajectories of comorbidity clusters are highlighted as key elements needed in current research in multimorbidity, so findings from this study should help to fill the knowledge gaps on multimorbidity in OA.

The relationship between chronic conditions and polypharmacy is a complex area of research. The count of the medications and more importantly the nature of prescribed drugs may be responsible for developing many new comorbidities in people with OA. We aim to explore the associations of the most commonly prescribed drugs in OA, such as NSAIDs, with the

For peer review only

incidence of a wide range of comorbidities, which will be the first time that conditions other than established comorbidities such as psychological conditions and endocrine diseases will be examined. Finally, the causality study will further explore the associations at genetic levels and phenotypes, which will be novel in OA research. Using a two sample MR approach - one for OA deep phenotypes and the other for other chronic conditions maximises the potentials of sample size, disease phenotypes and comorbidity spectrum to better explore the causal pathways between OA and comorbidity.

There are some limitations to this study. Firstly, there are inherent issues in the nature of electronic health records with respect to possible misdiagnosis, ascertainment biases, under- or over-recording, and changes in databases due to change in coding structures. Also, the analysis will be restricted to fewer covariates in some databases due to missing information on lifestyle factors such as physical activities and diet. Even though the databases have different durations of data available, if possible we will use a common follow-up time for objective five. Another important limitation is that we do not have information on quality of life and other outcomes to measure functional limitations recorded in the database. Chronic conditions, especially comorbidities recorded in general practices, depend on multiple factors such as population structure, health care facilities, health policies, and the nature of the national databases. A major strength of this study is that it will include medical records on approximately 27 million people in four European countries. Also, the study will cover the sequence of research questions in comorbidity or multimorbidity starting from the burden through to the causality and variation. Such a research model can be used for other similar multimorbidity studies. The expected results should inform health professionals in primary care settings with respect to management of people with OA and associated comorbidities.

Status of the study

All the centres have obtained the necessary approvals for using the database in 2020. A consensus has been made on the code mapping exercise. The statistical analysis will be explained in detail in each of the publications. The team is expected to produce results by mid-2021.

Acknowledgements: We thank the Patient Research Participants (PRP) members Jenny Cockshull, Stevie Vanhegan, and Irene Pitsillidou for their involvement since the beginning of the project. We would like to thank the FOREUM for financially supporting the research. The authors would like to acknowledge Keele University's Prognosis and Consultation Epidemiology Research Group who have given us permission to utilise the Code Lists (©2014).

For peer review only - http://bmjopen.bmj.com/site/about/guidelines.xhtml

Contributor and guarantor information:

WZ, MD, CC, SMA, ME, DA conceived and designed the study. SS, AK, AD, AT, DR, and VS developed the methods and will perform the analysis, and interpretation of the results. CC, WZ, JR, AS, CFK, VS, and AT will supervise the statistical analysis. CM and MD will guide with clinical interpretations of the results. SS drafted this manuscript and all authors contributed to the critical revision of the manuscript for important intellectual content. The corresponding author attests that all listed authors meet authorship criteria and that no others meeting the criteria have been omitted.

Funding: This work was supported by Foundation for Research in Rheumatology (FOREUM) grant (2019-2022), The Swedish Research Council (2020-01103), Governmental funding of clinical research within the national health services (ALF), and The Swedish Rheumatism Association. CM is funded by the National Institute for Health Research (NIHR) Applied Research Collaboration West Midlands, the National Institute for Health Research (NIHR) School for Primary Care Research and a National Institute for Health Research (NIHR) Research Professorship in General Practice (NIHR-RP-2014-04-026)

Role of the funding sources: The sponsors did not participate in the design and conduct of the study; collection, management, analysis, and interpretation of the data; or preparation, review, or approval of the manuscript and the decision to submit the manuscript for publication.

Competing interests: WZ declares serving as an advisory board for Ely Lilly (Ixekizumad, 2020) and Regeneron (Fasinomab, 2020). ME declares serving as an advisory Panel Board Member for Pfizer (Nov 2019, Tanezumab). CM provided advice to BMS on recruiting to a non-pharmacological atrial fibrillation trial. The other authors report no financial competing interests.

Copy right: The Corresponding Author has the right to grant on behalf of all authors and does grant on behalf of all authors, an exclusive licence (or non-exclusive for government employees) on a worldwide basis to the BMJ Publishing Group Ltd to permit this article (if accepted) to be published in BMJ editions and any other BMJPGL products and sublicences such use and exploit all subsidiary rights, as set out in our licence.

Studies involving humans or animals: No direct participant recruitment was done for the study.

Data sharing statement: We used anonymised data on individual patients on which the analysis, results, and conclusions reported in the paper are based. The used data is not distributable under licence. However, the relevant data can be obtained directly from the respective agencies. The codes developed for the analysis can be available upon a valid request.

Figure legend

Figure 1. Overview of the study design and statistical analysis plan for the Comorbidities in Osteoarthritis (CoMO) study.

For peer review only

For peer review only - http://bmjopen.bmj.com/site/about/guidelines.xhtml

Reference

1. Turkiewicz A, Petersson IF, Björk J, Hawker G, Dahlberg LE, Lohmander LS, et al. Current and future impact of osteoarthritis on health care: a population-based study with projections to year 2032. Osteoarthritis Cartilage. 2014 Nov;22(11):1826–32.

2. Badley EM. The effect of osteoarthritis on disability and health care use in Canada. J Rheumatol Suppl. 1995 Feb;43:19–22.

3. McDonough CM, Jette AM. The Contribution of Osteoarthritis to Functional Limitations and Disability. Clin Geriatr Med. 2010 Aug;26(3):387–99.

4. Cieza A, Causey K, Kamenov K, Hanson SW, Chatterji S, Vos T. Global estimates of the need for rehabilitation based on the Global Burden of Disease study 2019: a systematic analysis for the Global Burden of Disease Study 2019. The Lancet. 2020 Dec;396(10267):2006–17.

5. World Health Organization. Musculoskeletal conditions [Internet]. [cited 2021 Apr 6]. Available from: https://www.who.int/news-room/fact-sheets/detail/musculoskeletal-conditions

6. Swain S, Sarmanova A, Coupland C, Doherty M, Zhang W. Comorbidities in Osteoarthritis: A systematic review and meta-analysis of observational studies. 2019 Jun 17 [cited 2019 Jun 30]; Available from: http://doi.wiley.com/10.1002/acr.24008

7. Suri P, Morgenroth DC, Hunter DJ. Epidemiology of osteoarthritis and associated comorbidities. PM R. 2012 May;4(5 Suppl):S10-19.

8. Hall AJ, Stubbs B, Mamas MA, Myint PK, Smith TO. Association between osteoarthritis and cardiovascular disease: Systematic review and meta-analysis. Eur J Prev Cardiol. 2016;23(9):938–46.

9. Stubbs B, Aluko Y, Myint PK, Smith TO. Prevalence of depressive symptoms and anxiety in osteoarthritis: a systematic review and meta-analysis. Age Ageing. 2016 Mar 1;45(2):228–35.

10. Ricci-Cabello I, Stevens S, Kontopantelis E, Dalton ARH, Griffiths RI, Campbell JL, et al. Impact of the Prevalence of Concordant and Discordant Conditions on the Quality of Diabetes Care in Family Practices in England. Ann Fam Med. 2015 Nov 1;13(6):514–22.

11. Calderón-Larrañaga A, Poblador-Plou B, González-Rubio F, Gimeno-Feliu LA, Abad-Díez JM, Prados-Torres A. Multimorbidity, polypharmacy, referrals, and adverse drug events: Are we doing things well? Br J Gen Pract. 2012;62(605):e821–6.

12. Jüni P, Nartey L, Reichenbach S, Sterchi R, Dieppe PA, Egger M. Risk of cardiovascular events and rofecoxib: cumulative meta-analysis. The Lancet. 2004 Dec;364(9450):2021–9.

13. Castellsague J, Riera-Guardia N, Calingaert B, Varas-Lorenzo C, Fourrier-Reglat A, Nicotra F, et al. Individual NSAIDs and Upper Gastrointestinal Complications: A Systematic Review and Meta-Analysis of Observational Studies (the SOS Project). Drug Saf. 2012 Dec;35(12):1127–46.

14. Lowe D.B., Taylor M.J., Hill S.J. Cross-sectional examination of musculoskeletal conditions and multimorbidity: influence of different thresholds and definitions on prevalence and association estimates. BMC Res Notes. 2017;10(1):51.

15. John R, Kerby DS, Hagan Hennessy C. Patterns and Impact of Comorbidity and Multimorbidity Among Community-Resident American Indian Elders. The Gerontologist. 2003 Oct 1;43(5):649–60.

For peer review only - http://bmjopen.bmj.com/site/about/guidelines.xhtml

16. Alaeddini A., Jaramillo C.A., Faruqui S.H.A., Pugh M.J. Mining Major Transitions of Chronic Conditions in Patients with Multiple Chronic Conditions. Methods Inf Med. 2017;56(5):391–400.

17. Sudlow C, Gallacher J, Allen N, Beral V, Burton P, Danesh J, et al. UK Biobank: An Open Access Resource for Identifying the Causes of a Wide Range of Complex Diseases of Middle and Old Age. PLOS Med. 2015 Mar 31;12(3):e1001779.

18. Engelhart MJ, Geerlings MI, Meijer J, Kiliaan A, Ruitenberg A, van Swieten JC, et al. Inflammatory Proteins in Plasma and the Risk of Dementia: The Rotterdam Study. Arch Neurol. 2004 May 1;61(5):668.

19. Quality and Outcome Framework (QOF) [Internet]. [cited 2018 Mar 21]. Available from: https://digital.nhs.uk/article/8910/Quality-and-Outcome-Framework-QOF-Indicators-No-Longer-In-QOF-INLIQ-Enhanced-Services-ES-Vaccinations-and-Immunisations-V-I-and-GMS-Core-Contract-CC-extraction-specifications-business-rules-

20. Medicare C for, Baltimore MS 7500 SB, Usa M. CC_Main [Internet]. 2017 [cited 2018 Oct 24]. Available from: https://www.cms.gov/Research-Statistics-Data-and-Systems/Statistics-Trends-and-Reports/Chronic-Conditions/CC_Main.html

21. James SL, Abate D, Abate KH, Abay SM, Abbafati C, Abbasi N, et al. Global, regional, and national incidence, prevalence, and years lived with disability for 354 diseases and injuries for 195 countries and territories, 1990–2017: a systematic analysis for the Global Burden of Disease Study 2017. The Lancet. 2018 Nov;392(10159):1789–858.

22. Charlson ME, Pompei P, Ales KL, MacKenzie CR. A new method of classifying prognostic comorbidity in longitudinal studies: Development and validation. J Chronic Dis. 1987 Jan 1;40(5):373–83.

23. Sarmanova A, Fernandes GS, Richardson H, Valdes AM, Walsh DA, Zhang W, et al. Contribution of central and peripheral risk factors to prevalence, incidence and progression of knee pain: a community-based cohort study. Osteoarthritis Cartilage. 2018 Nov;26(11):1461–73.

24. Charlson M, Szatrowski TP, Peterson J, Gold J. Validation of a combined comorbidity index. J Clin Epidemiol. 1994 Nov 1;47(11):1245–51.

25. Quan H, Li B, Couris CM, Fushimi K, Graham P, Hider P, et al. Updating and Validating the Charlson Comorbidity Index and Score for Risk Adjustment in Hospital Discharge Abstracts Using Data From 6 Countries. Am J Epidemiol. 2011 Mar 15;173(6):676–82.

26. van Walraven C, Austin PC, Jennings A, Quan H, Forster AJ. A Modification of the Elixhauser Comorbidity Measures Into a Point System for Hospital Death Using Administrative Data: Med Care. 2009 Jun;47(6):626–33.

27. Elixhauser A, Steiner C, Harris DR, Coffey RM. Comorbidity measures for use with administrative data. Med Care. 1998 Jan;36(1):8–27.

28. Kuo C-F, Grainge MJ, Mallen C, Zhang W, Doherty M. Comorbidities in patients with gout prior to and following diagnosis: case-control study. Ann Rheum Dis. 2014 Nov 14;annrheumdis-2014-206410.

29. Swain S, Coupland C, Mallen C, Kuo CF, Sarmanova A, Bierma-Zeinstra SMA, et al. Temporal relationship between osteoarthritis and comorbidities: a combined case control and cohort study in the UK primary care setting. Rheumatology. 2021 Mar 1;keab067.

For peer review only - http://bmjopen.bmj.com/site/about/guidelines.xhtml

30. Collins LM, Lanza ST. Latent Class and Latent Transition Analysis [Internet]. Hoboken, NJ, USA: John Wiley & Sons, Inc.; 2009 [cited 2020 Apr 5]. (Wiley Series in Probability and Statistics). Available from: http://doi.wiley.com/10.1002/9780470567333

31. Proust-Lima C, Séne M, Taylor JM, Jacqmin-Gadda H. Joint latent class models for longitudinal and time-to-event data: A review. Stat Methods Med Res. 2014 Feb;23(1):74–90.

32. Vincent P, Larochelle H, Lajoie I, Bengio Y, Manzagol P-A. Stacked Denoising Autoencoders: Learning Useful Representations in a Deep Network with a Local Denoising Criterion. J Mach Learn Res. 2010 Dec;11:3371–408.

33. Zeng C, Zhang W, Doherty M, Persson MSM, Mallen C, Swain S, et al. Initial analgesic prescriptions for osteoarthritis in the United Kingdom, 2000–2016. Rheumatology. 2021 Jan 5;60(1):147–59.

34. Hippisley-Cox J, Coupland C. Unintended effects of statins in men and women in England and Wales: population based cohort study using the QResearch database. BMJ. 2010 May 20;340(may19 4):c2197–c2197.

35. Sarmanova A, Doherty M, Kuo C, Wei J, Abhishek A, Mallen C, et al. Statin use and risk of joint replacement due to osteoarthritis and rheumatoid arthritis: a propensity-score matched longitudinal cohort study. Rheumatology. 2020 Oct 1;59(10):2898–907.

36. Kuo C-F, Grainge MJ, Mallen C, Zhang W, Doherty M. Effect of allopurinol on all-cause mortality in adults with incident gout: propensity score–matched landmark analysis. Rheumatology. 2015 Jul 12;kev246.

37. Royston P, Lambert PC. Flexible parametric survival analysis using Stata: beyond the Cox model. College Station, TX: Stata Press; 2011. 347 p.

38. Millard LAC, Davies NM, Timpson NJ, Tilling K, Flach PA, Smith GD. MR-PheWAS: hypothesis prioritization among potential causal effects of body mass index on many outcomes, using Mendelian randomization. Sci Rep [Internet]. 2015 Dec [cited 2020 Jan 26];5(1). Available from: http://www.nature.com/articles/srep16645

39. Davies NM, Holmes MV, Davey Smith G. Reading Mendelian randomisation studies: a guide, glossary, and checklist for clinicians. BMJ. 2018 Jul 12;k601.

40. Li X, Meng X, Spiliopoulou A, Timofeeva M, Wei W-Q, Gifford A, et al. MR-PheWAS: exploring the causal effect of SUA level on multiple disease outcomes by using genetic instruments in UK Biobank. Ann Rheum Dis. 2018 Jul;77(7):1039–47.

41. Benjamini Y, Yekutieli D. The Control of the False Discovery Rate in Multiple Testing under Dependency. Ann Stat. 2001;29(4):1165–88.

42. Burgess S, Butterworth A, Thompson SG. Mendelian Randomization Analysis With Multiple Genetic Variants Using Summarized Data: Mendelian Randomization Using Summarized Data. Genet Epidemiol. 2013 Nov;37(7):658–65.

43. Bowden J, Davey Smith G, Haycock PC, Burgess S. Consistent Estimation in Mendelian Randomization with Some Invalid Instruments Using a Weighted Median Estimator. Genet Epidemiol. 2016 May;40(4):304–14.

For peer review only

44. Zhu Z, Zhang F, Hu H, Bakshi A, Robinson MR, Powell JE, et al. Integration of summary data from GWAS and eQTL studies predicts complex trait gene targets. Nat Genet. 2016 May;48(5):481–7.

45. Higgins JPT, Thompson SG. Quantifying heterogeneity in a meta-analysis. Stat Med. 2002 Jun 15;21(11):1539–58.

46. Stewart LA, Tierney JF. To IPD or not to IPD? Advantages and disadvantages of systematic reviews using individual patient data. Eval Health Prof. 2002 Mar;25(1):76–97.

47. Vetrano DL, Roso-Llorach A, Fernández S, Guisado-Clavero M, Violán C, Onder G, et al. Twelve-year clinical trajectories of multimorbidity in a population of older adults. Nat Commun [Internet]. 2020 Dec [cited 2020 Aug 12];11(1). Available from: http://www.nature.com/articles/s41467-020-16780-x

For peer review only

For peer review only - http://bmjopen.bmj.com/site/about/guidelines.xhtml

Table 1. Characteristics of the included databases

	Netherlands	Spain	Sweden	UK
Objectives 1-3				
Name of the database	Integrated Primary Care Information (IPCI)	The Spanish Sistema information del Deveolpment de l'Investigació a Atenció Primària (SIDIAP)	Skåne Healthcare Register	Clinical Practice Research Datalink (CPRD)
Settings	Routinely collected primary care database	Routinely collected primary care data	Swedish healthcare in Skane region, primary, specialist and in-patient care	Routinely collected primary care database with linkage database
Size and Coverage	2.2 million (Randomly distributed over the country)	6.5 million (> 85% of total Catalan region)	1.3 million (all residents of the Skane region)	17 million (country-wide, nearly 740 practices)
Start year	1998- (Better coding after 2000)	2006	1998	1993-
Age group	All	All	All	All
Gender	All	All	All	All
Coding system	ICPC	ICD 10	ICD 10	Read codes and ICD 10
Drug prescribed by	GP	GP	GP	GP
Death record (Either date of death and/or cause)	Both date and cause	Only date	Both date and cause (until year 2015)	Only date
Covariates/ additional variables	NA	BMI, Smoking, Alcohol, Social class, cholesterol, and other biomarkers	Education, Income, profession, and sick leave, residential area, region of birth	BMI, Smoking, Alcohol, Deprivation index, Ethnic group

BMI- Body mass index; GP- general practitioners; ICD- International classification of diseases; ICPC- International classification of primary care; NA- Not available

For peer review only

Table 2. Database for the mendelian randomisation

Name of the database	Rotterdam Cohort Study	UK Biobank
Population coverage	15000	500,000
Age group	>=40 years	40-69 years
Start year -till now	1989-onwards	2010-
Types of data	Radiographic data, joint pain, joint stiffness, of hip, knee, and hand, GWAS, biochemical markers	Genetic and phenotypes

GWAS- Genome Wide Association Studies

For peer review only

Table 3. List of chronic conditions across four databases

Sl no	Conditions	ICD 10	ICPC	Read Code
1	Anaemia (All types)	D50-D64	B78, B80, B81, B82	D00…, D01…
2	Ankylosing spondylitis	M45.9	NA	N10…
3	Anxiety disorder	F41.0-F41.9	P74, P74.01, P74.02	E200… , Eu41…
4	Asthma	J450-J45.9, J46.9	R96…	66Y…, H33…
5	Benign prostatic hypertrophy (BPH)	N40.9	Y85	K20…, K21…, K22…
6	Cardiac arrhythmias (Atrial Fibrillation)	I47.0-I49.9	K78	Gyu… , G573…
7	Cataract	H25.0-H25.9, H26.1-H26.9	F92	F46… ,
8	Chronic Back pain	M47-M48, M51-M54, M99, G54.4	L02, L03, L86	N12…, N14…,
9	Chronic kidney disease (any cause)	N02.0-N8.8, N11.0-N11.9, N12.9, N15.0-N18.9, N19.9	U99.01	1Z1… , K01…, K02…
10	Chronic neck pain	M54.2	L83…	Nyu… , N11…, N12… , N14…
11	Chronic sinusitis	J32 …	R75…	H13…
12	Chronic obstructive pulmonary diseases	J41.0-J41.8, J42.9, J43.0-J43.9, J44.0-J44.9	R91, R95	H3…
13	Coronary Heart Disease (Including Acute Myocardial infarction, Valvular disease, Angina),	I20.0-I25.0, I34.0-I37.0	K74-K76	G11…, G30…, G31… - G38..
14	Dementia	F00.0-F00.9, F01.0-F03.9, G30.0-G30.9, G31.0-G31.9	P70-P70.02	E00…, Eu0…, F11…
15	Depression	F32.0-F33.9	P76	Eu… , E11…
16	Diabetes mellitus	E10.0-E14.9	T90…, F83.01	C10…, F32…,
17	Dyslipidaemia (Hyper)	E78.1	T93	C32…
18	Eating disorders (Both)			Eu5… , R03…
19	Eczema/ Skin disease	L20.0-L22.9, L26.9	S74, S87, S88	M11…
20	Epilepsy	G40.0-G41.9	N88	F25…
21	Fatigue	F48.0	A04.11	F286…
22	Fibromyalgia	M79.7	L18.01	N248…, N239…
23	Gall bladder stone	K80.0-K80.8	D98-D98.03	781…, J65… , 4G2…,
24	GERD (Gastritis, Oesophageal bleeding, duodenitis, peptic ulcer)	K21 …	D840…	J12… , J13…, J15…
25	Gastrointestinal bleeding	K25.0-K28.9	D84-D87	J11… ,
26	Gout	M10.0-M10.9	T92	C34… , N023…
27	Hearing impairment (All types)	H90.0-H91.9	H83-H86	F59… , ZE87…
28	Heart Failure	150.0-150.9	K77-K77.02	G58…,
29	Hepatitis	K73.0-K73.9	D72-D72.05	J61… , J63…
30	HIV/AIDS	B20-B24	B90	A788… , A789…, AyuC…
31	Hypertension	I10.9, I11.0-I13.9, I15.0-I15.9	K86-K87, F83.02	G20…, G24…, G25…, G26…
32	Hyperthyroidism	E05.0-E05.9	T85	C02…
33	Hypothyroidism	E02.9, E03.0-E03.9	T86	C03…, C04…
34	Inflammatory Bowel Disease (IBD)	K50.0-K52.9	D94-D94.02	J4… ,
35	Irritable Bowel Symptoms (IBS)	K58.1-K58.8		J52…
36	Leukaemia, Lymphoma	C81.0-C86.6, C91.0-C96.9	B72-B73	B60… , B61…, B64…
37	Liver Cirrhosis	K70.0-K71.9, K74.0-K74.6	D97…	J615…
38	Migraine	G43.0-G43.9	N89	F26… ,
39	Multiple sclerosis	G35.9	N86	F20…,
40	Osteoarthritis	M16.0-M16.9, M17.0-M17.9	L89-L91	N05… ,
41	Osteoporosis	M80.0-M82.9	L95…	N33… ,

For peer review only - http://bmjopen.bmj.com/site/about/guidelines.xhtml

42	Other blood vessel disease (Raynaud's disease, Burger's disease)	I73.0-I73.9	K92…	G73… ,
43	Parkinson's disease	G20.9	N87-N87.01	F12…,
44	Peripheral vascular disease (Atherosclerosis)	170.0-170.9	K91	G70…, G71… , G72…
45	Polymyalgia	M35.3	L99.12	N20.11
46	Psoriasis	L40.0-L41.9	S91	M161…
47	Psoriatic arthritis	M07.0-M07.3	L99.13	M160…
48	Rheumatoid Arthritis	M05.0-M05.9	L88… , K71…	N04….
49	Renal stones	N20.0 ….	U95	4G4… , 7B07… , KB12…
50	Schizophrenia and/or psychosis	F20.0-F20.9, F25.0- F25.9	P72	E10…
51	Severe allergy			H17… , SN5…
52	Sjögren's syndrome	M35.0	NA	N002…
53	Systemic Lupus Erythematosus	M32.0, M32.1, M32.8, M32.9	NA	N000…
54	Sleep disorder (Insomnia)	F51.0	P06	Fy0… , 1B1B…
55	Solid malignancy	C00.0-C80.9, D00.0-D09.9, C97.9	A29… , A79, B74 – Y78	B0… - B67… , Byu…
56	Stroke	G45.0-G46.8, I60.0-I63.9, I65.0-166.9, I69.0-I69.4	K89-K90.02	G60… - G68…, F22…
57	Substance abuse/ Drug addiction	F10.0-F19.9	P18, P19…	E24…, Eu1…
58	Thrombotic diseases	I74.0-I74.9	K93, K94…, W99.03	G80… , G81… , G74…
59	Tuberculosis	A15.0-A16.9, B90.9	A70, R70	A1…, A11…
60	Vertigo	H81.4 …	N17-N17.02, H82-H82.03	R004… , F561…
61	Vision problem (Glaucoma and other)	H27.0-H27.9, H40.0-H40.9, H42.0-H42.8	F93…, F94	F45…, F49… ,

ICD- International Classification of Diseases; ICPC- International classification in primary care. All the codes are the primary code initials used in the database.

For peer review only - http://bmjopen.bmj.com/site/about/guidelines.xhtml

Table 4. Group of conditions/Outcome

Group	Conditions
Cardiovascular	Cardiac arrhythmias, Coronary Heart Disease (including AMI, valvular disease, angina), Heart failure, Hypertension, Peripheral vascular disease (claudication, Raynaud syndrome, Buerger's disease), Other blood vessel disease (atherosclerosis and aneurysm), Thrombotic diseases
Gastrointestinal	GERD (Esophageal diseases, gastritis, duodenitis), GI bleeding, Inflammatory bowel disease (IBD), Irritable bowel syndrome (IBS)
Musculoskeletal	Ankylosing spondylitis, Chronic Back pain, Chronic Neck pain, Fibromyalgia, Polymyalgia, Gout, Osteoporosis, Psoriatic arthritis, Rheumatoid arthritis, Sjögren's syndrome, Systemic lupus erythematosus (SLE)
Endocrine	Diabetes mellitus, Dyslipidemia (hyper), Hyperthyroidism, Hypothyroidism
Neurological	Dementia, Epilepsy, Fatigue, Migraine, Multiple sclerosis, Parkinson disease, Stroke
Psychological	Anxiety, Depression, Eating disorders (Anorexia / Bulimia nervosa), Schizophrenia, Sleep disorder (insomnia),
Kidney disease	Chronic kidney disease (any cause), Renal stones
Liver diseases	Gall bladder stone, Hepatitis, Liver cirrhosis
Respiratory	Asthma, Chronic obstructive pulmonary disease (COPD)
Cancer	Leukemia, Lymphoma, Solid Malignancy (any type)
Others	Anemia (all types), Benign prostate hypertrophy (BPH), Cataract, Chronic sinusitis, Eczema/Skin disease, Hearing impairment (all types), Psoriasis, Severe allergy (anaphylactic shock), angioneurotic oedema

For peer review only

For peer review only - http://bmjopen.bmj.com/site/about/guidelines.xhtml

Figure 1. Overview of the study design and statistical analysis plan for the Comorbidities in Osteoarthritis (CoMO) study.

	Objective 1	Objective 2	Objective 3	Objective 4
Study Design	Case-control and Cohort	Clustering and longitudinal	Cohort	Genomic association
Exposure	OA	OA	Analgesics (NSAIDS, Opioids, paracetamol)	OA
Outcome	Comorbidities	Clusters of comorbidities	Comorbidities	Comorbidities
Statistical methods	Conditional logistic regression,<br>Cox regression	Latent class analysis,<br>Latent class growth analysis<br>Joint latent class analysis	Cox regression<br>Time varying analysis<br>Flexible parametric method	Mendelian randomisation
Reported Outcome	Odds Ratio and Hazard Ratio	Clusters and groups	Hazard Ratio	Coefficients
Participating centres	ALL	ALL	ALL	UK and Netherlands

Objective 5 — **Meta Analysis**

OA- osteoarthritis; NSAIDs- Nonsteroidal anti-inflammatory drugs

For peer review only

STROBE 2007 (v4) checklist of items to be included in reports of observational studies in epidemiology*

Checklist for cohort, case-control, and cross-sectional studies (combined)

**Section/Topic**	**Item #**	**Recommendation**	**Reported on page #**
**Title and abstract**	1	**(*a*) Indicate the study's design with a commonly used term in the title or the abstract**<br>Comorbidities in Osteoarthritis (CoMO): a multinational study in four European countries	1-2
		**(*b*) Provide in the abstract an informative and balanced summary of what was done and what was found**<br>*Introduction*<br>Osteoarthritis (OA) is one of the leading chronic conditions in the older population. People with OA are more likely to have one or more other chronic conditions than those without. However, the temporal associations, clusters of the comorbidities, role of analgesics and the causality and variation between populations are yet to be investigated. This paper describes the protocol of a multinational study in four European countries (UK, Netherlands, Sweden, and Spain) exploring comorbidities in people with OA .<br>*Methods and analysis*<br>This multinational study will investigate i) the temporal associations of 61 identified comorbidities with OA, ii) the clusters and trajectories of comorbidities in people with OA, iii) the role of analgesics on incidence of comorbidities in people with OA, iv) the potential biomarkers and causality between OA and the comorbidities, and v) variations between countries.<br>A combined case-control and cohort study will be conducted to find the temporal association of OA with the comorbidities using the national or regional health databases. Latent class analysis will be performed to identify the clusters at baseline and joint latent class analysis will be used to examine trajectories during the follow-up. A cohort study will be undertaken to evaluate the role of non-steroidal anti-inflammatory drugs (NSAIDs), opioids, and paracetamol on the incidence of comorbidities. Mendelian randomisation will be performed to investigate the potential biomarkers for causality between OA and the comorbidities using the UK Biobank and the Rotterdam	1

For peer review only

		Study databases. Finally, a meta-analyses will be used to examine the variations and pool the results from different countries.<br>*Ethics and Dissemination*<br>Research ethics was obtained according to each database requirement. Results will be disseminated through the FOREUM website, scientific meetings, publications, and in partnership with patient organisations.	
**Introduction**			
Background/rationale	2	**Explain the scientific background and rationale for the investigation being reported**<br>Osteoarthritis (OA) affects 27% of people aged over 45 years at peripheral synovial joints such as knees, hips, hands and feet (1). It is by far the most common form of arthritis, and a leading cause of chronic joint pain and disability in older people (2,3). It is anticipated that the burden of OA will continue to rise in the coming decades because of population ageing and the increasing obesity prevalence – two major risk factors for OA (4,5). Co-occurrence of multiple chronic conditions in an individual with ageing is becoming a norm and OA is not an exception to this.<br>A recent systematic review has confirmed that people with OA are more likely to have other diseases, especially stroke, peptic ulcer, hypertension, and depression (6). Vast majority of these studies focused on additional presence (comorbidity) of cardiovascular and musculoskeletal conditions only (7–9). Whether these comorbidities just co-exist with OA, share common risk factors with OA, or are causes or consequences of OA remains largely unknown. There was also reporting of wide heterogeneity in definitions of OA and other chronic conditions, diagnosis and recording of diseases, sample sizes, and number of diseases studied in previously published studies included in the review (6). This diversity made the comparison and pooled estimation of comorbidity prevalence difficult.<br>Comorbidity in OA can occur due to multiple factors. Various hypotheses have been used to explain the existence of comorbidities in general, the most accepted of which are the concordant (diseases sharing similar	4-5

For peer review only

		pathophysiological risk factors) and discordant (diseases not sharing similar pathophysiological risk factors) theories (10). Additionally, prescription of drugs is also reported to be associated with comorbidity and multimorbidity (11). Especially in people with OA, the prescription of analgesics is common, and is associated with increased risk of other conditions such as cardiovascular, gastrointestinal and chronic renal diseases (12,13). Also, having multiple chronic conditions increases the chances of polypharmacy which further escalates the risk of other conditions.<br>OA is one of the leading conditions reported in multimorbidity research. Exploring the association of OA with other diseases would help in further explaining the burden and pattern of the comorbidity (14). However, the major issue in OA comorbidity research is the low number and specific types of conditions studied (16). Therefore, it is important to develop a consensus on both the count and typology of conditions to be studied to enable comparisons across populations and to derive pooled estimates as appropriate. Further, using uniform methods and definitions of diseases in computing these estimates would reduce heterogeneity and make the comparison more reliable. Understanding the temporal association with comorbidity and disease trajectory is crucial for any chronic condition, and this is possible through studies using longitudinal databases (15). However, one of the limitations of using observational data is that causal associations are difficult to establish, due to the interference of known and unknown confounders. In this study we have used the more recently developed method ‘Mendelian Randomization’, that can determine causal estimates through combining the use of genetic data and instrumental variable methods.”<br>The burden of diseases in primary care often depends on the population structure, health infrastructure and individual factors such as income and education. Such factors vary between countries, because of the heterogeneity mentioned above there are no robust data comparing OA and its comorbidities between countries. Therefore, the aims of this study were to explore the burden, pattern, and causal factors of comorbidities in people with OA across four European countries using national registration databases in the UK, the Netherlands, Sweden, and	

For peer review only

		Spain.	
Objectives	3	**State specific objectives, including any pre-specified hypotheses**<br>1. To estimate the prevalence, incidence, and time sequence of comorbidities in OA<br>2. To examine the clusters of comorbidities and trajectories of clusters in OA and associations with death<br>3. To investigate the associations between commonly used OA drugs, such as non-steroidal anti-inflammatory (NSAIDs) and opioids, and risk of comorbidities<br>4. To identify the potential biomarkers and causal pathways between OA and the comorbidities<br>5. To examine the variations of OA comorbidities and clusters across countries.	5
**Methods**			
Study design	4	**Present key elements of study design early in the paper**<br>Figure 1	Figure 1
Setting	5	**Describe the setting, locations, and relevant dates, including periods of recruitment, exposure, follow-up, and data collection**<br>*Databases*<br>Four routinely collected national (the UK and the Netherlands) or regional (Sweden and Spain) health databases will be used for objectives 1-3. In addition, for objective 4, genomic associations of OA with comorbidities will be examined using two cohort studies from the UK (UK Biobank) (17) and the Netherlands (Rotterdam study) (18). The four national representative and regional databases contain information about the population with primary care consultations in four different countries. The longitudinal databases provide information about the diagnosis of the diseases by the general practitioners and some diagnoses made in secondary care, prescription of drugs, deaths, and other health utilisation indicators. The details of the databases and their properties are given in Table 1 and 2.<br>*Participants*<br>People registered with the respective databases aged 18 year or above are eligible for the study.	5-6

Participants	6	(*a*) *Cohort study*—Give the eligibility criteria, and the sources and methods of selection of participants. Describe methods of follow-up<br>*Case-control study*—Give the eligibility criteria, and the sources and methods of case ascertainment and control selection. Give the rationale for the choice of cases and controls	n/a
		**(*b*) *Cohort study*—For matched studies, give matching criteria and number of exposed and unexposed**<br>***Case-control study*—For matched studies, give matching criteria and the number of controls per case**<br><br>*Objective 1 – Prevalence, incidence, and time sequence of comorbidities in OA*<br><br>A combined retrospective and prospective study of OA cases and sex, age (+/-2 years), first year of registration, and practice matched controls (1:1-4) without OA (28) will be used to determine the prevalence, incidence, and time sequence of comorbidities in OA. Incident OA cases will be identified, and the first diagnosis date will be used as the starting point (index date). For controls the same index date as their matched case will be used. They will be both retrospectively reviewed for prior diagnoses of comorbidities and prospectively followed-up for posterior new comorbidities.<br>For the prospective analysis participants with incident OA but without the specific comorbidity of interest at the index date (i.e., people at risk) and matched controls without OA will be followed up until the date of the first diagnosis of the comorbidity, deregistration, or death whichever comes first. The cumulative incidence will be calculated for each comorbidity in OA cases and matched controls at 1, 3, 5, 10, 15, 20 years (based on the data available) after the index date to examine the dynamic change of developing comorbidities during follow-up.<br>*Objective 3*<br>A cohort study will be undertaken for this objective to evaluate the contribution of common analgesics for OA to the development of comorbidity such as NSAIDs, opioids and paracetamol. We are interested in the interaction between OA and use of drugs on the incidence of comorbidities, i.e. to evaluate if the drug use in persons with OA poses increased or decreased risk of comorbidities compared to persons without OA and/or analgesics. Individual	7-8<br><br>9

For peer review only

		comorbidity, as well as clusters of comorbidities identified from Objective 2 will be examined as outcomes. The 61 comorbidities in our study will be further categorised into eight groups, specifically: musculoskeletal (MSK), respiratory, neurodegenerative, psychological/psychiatric, cancer, cardiovascular, metabolic, renal problem, liver diseases, gastrointestinal (GI) and others. (Table 4) The prospective cohort established from Objective 1 will form the source population for this objective. Individuals with incident OA will be identified from the database and the first diagnosis date will be used as index date for follow-up. Individuals without OA during the study period will be selected and matched with cases by age, sex, and practice. The same index date will be given from their matched OA cases. Individuals with analgesics prescriptions prior to the index date will be excluded (or recorded as a confounding factor to be adjusted as appropriate). Only analgesic prescriptions after the index date will be considered for this analysis. Prescriptions will be quantified as number of prescriptions within year 1 (initial use, primary analysis) (33), 2, 3, 4, 5 etc. It will also be dichotomised as episodic (e.g., at least one gap of ≥90 days between prescriptions) and continuous (no gap of more less than 90 days) users as appropriate (34,35). Analgesic use will be included in the model as a risk factor together with OA diagnosis (yes/no, primary exposure) to examine the independent risk of each variable (OA and analgesics), as well as the interaction between the two to the development of comorbidity. Dose response relationship will be examined using number of prescriptions during the exposure window examined. The effect of stopping analgesics will also be examined by looking into the patterns of analgesic prescriptions, e.g., stopping analgesics after initial use in year 1 versus continuous use of analgesics afterwards.<br>*Objective 4*<br>We will use the Rotterdam Study and the UK Biobank jointly for this objective. This is because that the Rotterdam Study is an OA cohort with deep phenotypes and biomarkers of OA, whereas the UK Biobank is a primary cohort for cancer and multiple disease outcomes, and both have detailed genetic variants. We will use two sample MR	10

For peer review only

		approach, i.e., to establish an association between OA and genetic variants in the Rotterdam Cohort to identify genetic instrumental variables (IV), e.g., a set of single nucleotide polymorphisms (SNPs) associated with OA (or a deep phenotype, biomarker, or risk factor of OA). We will then undertake the MR-PheWAS analysis to examine the causal effects of the OA IV on comorbidities in the UK Biobank. The MR method has been widely used in real world data to examine the causal relationship between IV and specific disease, under two assumptions: [1] genetic variants are randomly assigned in the population; and [2] genetic variants can only be the cause not consequence of disease (39). The PheWAS is a series of case control studies to estimate the associations between the IV and multiple disease outcomes (38,40). The combination of the two permits investigation of the causal effects of OA on multiple disease outcomes.	
Variables	7	**Clearly define all outcomes, exposures, predictors, potential confounders, and effect modifiers. Give diagnostic criteria, if applicable**<br>*Definition of Osteoarthritis*<br>OA will be defined as having at least one recorded physician diagnosis of OA for hip, knee, ankle/foot, wrist/hand, or site recorded as 'unspecified' during the study period for the respective database. People with any previous recording of the OA prior to the start date of the study will be excluded.<br>*Comorbidities*<br>We defined comorbidity as the recording of diagnosis of predefined chronic conditions in individuals using either ICD-10 or Read or international classification of primary care (ICPC) code. An extensive list of 61 chronic conditions was prepared from the Quality Outcome Framework (QOF) (19), list of the US Department of Health and Human Services Initiative on Multiple Chronic Conditions (20), global burden of diseases (21) and the Charlson comorbidity index (22). The list has been updated with findings from our systematic review (6) and a previous UK community-based knee pain study (6,23) by including common and important morbidities not included in the above (24,25). A code mapping exercise was conducted to finalise the list of conditions available for all the research centres. The comparison of codes was made, and it was reviewed by four researchers including	6-7

		a clinician from the team. The detailed list of the conditions is given in Table 3.<br><br>*Covariates*<br>Age and sex will be used in all centres as covariates to adjust in regression models. Additionally, information on body mass index (BMI), smoking, alcohol use, socioeconomic variables such as education level, income, place of birth (to identify those who immigrated to the country), and residential area, marriage (or registered partner) will be included when available. For calculating severity of the comorbidities in an individual, Elixhauser comorbidity index will be used to estimate the impact of comorbidities on death (26,27). Missing data on covariates will be substituted using multiple imputation methods, provided that the data is missing at random, if applicable.	
Data sources/ measurement	8*	**For each variable of interest, give sources of data and details of methods of assessment (measurement). Describe comparability of assessment methods if there is more than one group**<br>*Databases*<br>Four routinely collected national (the UK and the Netherlands) or regional (Sweden and Spain) health databases will be used for objectives 1-3. In addition, for objective 4, genomic associations of OA with comorbidities will be examined using two cohort studies from the UK (UK Biobank) (17) and the Netherlands (Rotterdam study) (18). The four national representative and regional databases contain information about the population with primary care consultations in four different countries. The longitudinal databases provide information about the diagnosis of the diseases by the general practitioners and some diagnoses made in secondary care, prescription of drugs, deaths, and other health utilisation indicators. The details of the databases and their properties are given in Table 1 and 2.<br><br>*Data Harmonisation:*<br>Firstly, we carried out a code mapping exercise for identification of people with osteoarthritis (OA) and other comorbidities. We developed a list of chronic conditions and each centres shared the list of codes to be used for the conditions, such as Read code in CPRD (UK), ICPC2 (Rotterdam) and ICD-10 for Lund and Spanish as per their database. The code lists were compared and edited to maintain the uniformity. The list was screened by verified by	*5*<br><br>*7*

For peer review only

		two researchers and two GPs. We also used unform definition for inclusion of condition e.g. at least one recording of the chronic conditions. Because all the centres did not have all the listed comorbidities, a minimum number of chronic conditions and covariates common in all the database were identified to be studied. Similarly, we decided to have a minimum follow-up study of 5 years and centres with more registration period can use the entire length of data available.	
Bias	9	Describe any efforts to address potential sources of bias	Explained under each objective
Study size	10	Explain how the study size was arrived at<br>*Feasibility and sample size*<br>To detect minimum incidence of 1% comorbidity (required for cluster analysis) with a minimum clinical important difference of hazard ratio (HR)1.2, and 90% power of the study, the estimated sample size was 197561 for 1581 events. It was calculated using STATA, with a correlation= 0.2, standard deviation of 0.5, proportion of withdrawal= 0.20, alpha=0.05. The initial check with the registry database revealed to have minimum required sample size for the study.	11-12
Quantitative variables	11	Explain how quantitative variables were handled in the analyses. If applicable, describe which groupings were chosen and why	Explained under each objective
Statistical methods	12	**(*a*) Describe all statistical methods, including those used to control for confounding**<br>*Objective 1*<br>In the retrospective analysis the prevalence and 95% confidence interval (CI) of each specific comorbidity will be calculated separately in OA cases and matched controls using the number of people diagnosed with the comorbidity divided by the total number of OA cases or controls at the index date. The prevalence of each	8

For peer review only

		comorbidity in OA cases and matched controls will be calculated for given time intervals prior to the index date of 0-1, 0-5 and 0-10 years separately to assess observational bias (28). Discrete time intervals of 1-5, and 5-10 years before will also be used to estimate the prevalence to minimise consultation bias/misclassification bias of OA (if possible). Logistic regression will be used to calculate the odds ratios (OR) for each comorbidity unadjusted and adjusted for BMI, smoking and alcohol consumption.<br>Kaplan-Meier survival curves will be used to display the cumulative probability in OA and non-OA groups. Proportional hazard assumption will be tested using Schoenfeld residual plots. The Cox regression model will be used to calculate hazard ratios (HR) for each comorbidity unadjusted and adjusted for age, sex, practice, BMI, smoking and alcohol consumption. This hybrid design has been previously used by us to examine the temporality of associations between other rheumatic musculoskeletal diseases (RMDs) (e.g., gout and lupus) and comorbidities (28,29).	8
		Objective 2<br><br>For each dataset, an 80%: 20% split into the training and testing data will be introduced. The following analysis in objective 2 will be first employed into the training dataset and then tested its generalisability in the testing dataset. At baseline, clusters of people based on 61 comorbidities will be identified using Latent class (i.e., Gaussian mixture models algorithms of cluster) analysis (30). For each model, we will examine the association between clusters and covariates using multinomial logistic regressions. The distinctness of clusters will be examined by comparing covariates among clusters. The optimal model is the one where most clusters found in the training data are also identified independently in the testing data and clusters have most distinct patients' characteristics. We will then use both latent trajectory analysis, such as joint latent class models (31), and unsupervised machine learning approach, such as deep autoencoder or recurrent neural networks (32), to identify distinct clusters of new	9

For peer review only

		comorbidity numbers development over time and their association with mortality with adjustment for baseline covariates.<br>*Objective 3*	
		For the primary analysis (initial prescriptions within year 1), a landmark analysis will be used to minimise the immortal time bias where the follow up will start after 12 months from the index date (36). Participants at risk (i.e., without a specific comorbidity of interest) at the landmark date will be followed up until the first diagnosis of the comorbidity, deregistration, or death whichever comes first. For secondary analyses, time varying covariate analysis will be used to examine the long-term, episodic/continuous use of analgesics after the index date and interaction between OA and analgesics in the development of the comorbidity. The propensity score matching or the inverse probability weighing methods will be used to adjust for confounding by indication during the follow-up as appropriate. Depending on the country-specific drug use patterns, we may modify this definition to allow for short brakes in between the episodes. Cox-regression model will be used to calculate the HR and 95% CI. We will use flexible parametric models using restricted cubic splines (developed by Lambert, "stpm2" in Stata) to estimate the HRs and differences in time to diagnosis of comorbidities (outcome) with drugs as time-varying to account for non-proportional hazards (37).<br>*Objective 4*	10
		The MR-PheWAS analysis includes three steps. Firstly, we will identify the genetic variants that are associated with OA - IV. Secondly, we will undertake the PheWAS analysis – a series of case control analyses to estimate the associations between the IV and other disease outcomes (38,40) , with an adjustment for multiple testing using the false discovery rate (FDR) methods (41). Thirdly, we will implement conventional MR analysis to investigate the causal effects of the OA IV on comorbidities (39). An inverse variance weighted (IVW) method will be used to pool the associations (ORs) as appropriate (42). The MR-Egger regression analysis will be used to count for the pleiotropic effect - the effects of one genetic variant on multiple outcomes (43). The heterogeneity in dependent	10-11

For peer review only

		instruments (HEIDI) test will be used to exclude the cross-phenotype associations caused by genetic linkage (44).<br>With the MR-PheWAS study, the OR can be interpreted as causal association. We are primarily interested in the causality from OA to comorbidities. We are also interested in inflammatory (e.g., CRP), metabolic (e.g., gut microbiome) and biomechanics (e.g., BMI) biomarkers and deep phenotypes of OA such as knee, hip, and hand OA with and without symptoms. This will be undertaken if it is feasible within 3 years of this funded project, otherwise will be considered as our future research agenda.<br>*Objective 5*<br>We will use meta-analyses (MA) to examine the variation between countries and to pool the data as appropriate. Estimates from first three objectives such as prevalence, incidence, OR, HR and 95% CI for each specific comorbidity across different populations will be distributed in a forest plot. Heterogeneity will be examined using the I^2 statistic and the Q test (45). Results will be pooled if they are homogenous based on the I^2 value using the fixed effects model, otherwise the reasons for the heterogeneity will be investigated. Random effects models will be used to pool the results if the reasons for the heterogeneity cannot be identified and if the overall pooling is appropriate. Individual patient data (IPD) meta-analysis may be used to help identify the reasons for heterogeneity (46). Common clusters and trajectories as well as burdens of comorbidities will also be compared between populations.	11
		**(*b*) Describe any methods used to examine subgroups and interactions**<br>Please see method sections	n/a
		**(*c*) Explain how missing data were addressed**<br>Missing data on covariates will be substituted using multiple imputation methods, provided that the data is missing at random, if applicable.	7
		(*d*) *Cohort study*—If applicable, explain how loss to follow-up was addressed<br>*Case-control study*—If applicable, explain how matching of cases and controls was addressed<br>*Cross-sectional study*—If applicable, describe analytical methods taking account of sampling strategy	n/a
		(*e*) Describe any sensitivity analyses	n/a

For peer review only

**Results**			
Participants	13*	(a) Report numbers of individuals at each stage of study—eg numbers potentially eligible, examined for eligibility, confirmed eligible, included in the study, completing follow-up, and analysed	n/a
		(b) Give reasons for non-participation at each stage	n/a
		(c) Consider use of a flow diagram	n/a
Descriptive data	14*	(a) Give characteristics of study participants (eg demographic, clinical, social) and information on exposures and potential confounders	n/a
		(b) Indicate number of participants with missing data for each variable of interest	n/a
		(c) *Cohort study*—Summarise follow-up time (eg, average and total amount)	n/a
Outcome data	15*	*Cohort study*—Report numbers of outcome events or summary measures over time	n/a
		Case-control study—Report numbers in each exposure category, or summary measures of exposure	n/a
		Cross-sectional study—Report numbers of outcome events or summary measures	n/a
Main results	16	(*a*) Give unadjusted estimates and, if applicable, confounder-adjusted estimates and their precision (eg, 95% confidence interval). Make clear which confounders were adjusted for and why they were included	n/a
		(*b*) Report category boundaries when continuous variables were categorized	n/a
		(*c*) If relevant, consider translating estimates of relative risk into absolute risk for a meaningful time period	n/a
Other analyses	17	Report other analyses done—eg analyses of subgroups and interactions, and sensitivity analyses	n/a
**Discussion**			
Key results	18	Summarise key results with reference to study objectives	n/a
Limitations	19	**Discuss limitations of the study, taking into account sources of potential bias or imprecision. Discuss both direction and magnitude of any potential bias**<br>There are some limitations to this study. Firstly, there are inherent issues in the nature of electronic health records with respect to possible misdiagnosis, ascertainment biases, under- or over-recording, and changes in databases due to change in coding structures. Also, the analysis will be restricted to fewer covariates in some databases due to missing information on lifestyle factors such as physical activities and diet. Even though the databases have different durations of data available, if possible we will use a common follow-up time for objective five. Another important limitation is that we do not have information on quality of life and other outcomes to measure functional limitations recorded in the database.<br>Chronic conditions, especially comorbidities recorded in general practices, depend on multiple factors such as population structure, health care facilities, health policies, and the nature of the national databases. A major	13

For peer review only

		strength of this study is that it will include medical records on approximately 27 million people in four European countries. Also, the study will cover the sequence of research questions in comorbidity or multimorbidity starting from the burden through to the causality and variation. Such a research model can be used for other similar multimorbidity studies. The expected results should inform health professionals in primary care settings with respect to management of people with OA and associated comorbidities.	
Interpretation	20	Give a cautious overall interpretation of results considering objectives, limitations, multiplicity of analyses, results from similar studies, and other relevant evidence	n/a
Generalisability	21	Discuss the generalisability (external validity) of the study results	n/a
**Other information**			
Funding	22	**Give the source of funding and the role of the funders for the present study and, if applicable, for the original study on which the present article is based** This work was supported by Foundation for Research in Rheumatology (FOREUM) grant (2019-2022), The Swedish Research Council (2020-01103), Governmental funding of clinical research within the national health services (ALF), and The Swedish Rheumatism Association. CM is funded by the National Institute for Health Research (NIHR) Applied Research Collaboration West Midlands, the National Institute for Health Research (NIHR) School for Primary Care Research and a National Institute for Health Research (NIHR) Research Professorship in General Practice (NIHR-RP-2014-04-026)	14

*Give information separately for cases and controls in case-control studies and, if applicable, for exposed and unexposed groups in cohort and cross-sectional studies.
Note: An Explanation and Elaboration article discusses each checklist item and gives methodological background and published examples of transparent reporting. The STROBE checklist is best used in conjunction with this article (freely available on the Web sites of PLoS Medicine at http://www.plosmedicine.org/, Annals of Internal Medicine at http://www.annals.org/, and Epidemiology at http://www.epidem.com/). Information on the STROBE Initiative is available at www.strobe-statement.org.

For peer review only
